# LncRNA CSMD1-1 promotes the progression of Hepatocellular Carcinoma by activating MYC signaling

**DOI:** 10.7150/thno.45989

**Published:** 2020-06-12

**Authors:** Ji Liu, Rui Xu, Shi-Juan Mai, Yu-Shui Ma, Mei-Yin Zhang, Ping-Sheng Cao, Nuo-Qing Weng, Rui-Qi Wang, Di Cao, Wei Wei, Rong-Ping Guo, Yao-Jun Zhang, Li Xu, Min-Shan Chen, Hui-Zhong Zhang, Long Huang, Da Fu, Hui-Yun Wang

**Affiliations:** 1 State Key Laboratory of Oncology in South China, and Collaborative Innovation Center for Cancer Medicine, Sun Yat-Sen University Cancer Center, Guangzhou, China 510060.; 2 Affiliated Cancer Hospital & Institute of Guangzhou Medical University, Guangzhou, China 510095.; 3 Central Laboratory for Medical Research, Shanghai Tenth People's Hospital, Tongji University School of Medicine.; 4 Department of Hepatobiliary Surgery, Sun Yat-Sen University Cancer Center, Guangzhou, China 510060.; 5 Department of Pathology, Sun Yat-Sen University Cancer Center, Guangzhou, China 510060.; 6 Department of Oncology, The Second Affiliated Hospital of Nanchang University, Nanchang, China.

**Keywords:** Long noncoding RNA, lncCSMD1-1, Hepatocellular carcinoma, MYC, prognosis

## Abstract

Emerging evidence suggests that long non-coding RNAs (lncRNA) play critical roles in the development and progression of diverse cancers including hepatocellular carcinoma (HCC), but the underlying molecular mechanisms of lncRNAs that are involved in hepatocarcinogenesis have not been fully explored.

**Methods:** In this study, we profiled lncRNA expression in 127 pairs of HCC and nontumor liver tissues (a Discovery Cohort) using a custom microarray. The expression and clinical significance of lncCSMD1-1 were then validated with qRT-PCR and COX regression analysis in a Validation Cohort (n=260) and two External Validation Cohorts (n=92 and n=124, respectively). In vitro and *in vivo* assays were performed to explore the biological effects of lncCSMD1-1 on HCC cells. The interaction of lncCSMD1-1 with MYC was identified by RNA pull-down and RNA immunoprecipitation. The role of LncCSMD1-1 in the degradation of MYC protein was also investigated.

**Results:** With microarray, we identified a highly upregulated lncRNA, lncCSMD1-1, which was associated with tumor progression and poor prognosis in the Discovery Cohort, and validated in another 3 HCC cohorts. Consistently, ectopic expression of lncCSMD1-1 notably promotes cell proliferation, migration, invasion, tumor growth and metastasis of HCC cells in *in vitro* and *in vivo* experiments. Gene expression profiling on HCC cells and gene sets enrichment analysis indicated that the MYC target gene set was significantly enriched in HCC cells overexpressing lncCSMD1-1, and lncCSMD1-1 was found to directly bind to MYC protein in the nucleus of HCC cells, which resulted in the elevation of MYC protein. Mechanistically, lncCSMD1-1 interacted with MYC protein to block its ubiquitin-proteasome degradation pathway, leading to activation of its downstream target genes.

**Conclusion:** lncCSMD1-1 is upregulated in HCC and promotes progression of HCC by activating the MYC signaling pathway. These results provide the evidence that lncCSMD1-1 may serve as a novel prognostic marker and potential therapeutic target for HCC.

## Introduction

Hepatocellular carcinoma (HCC) constitutes the majority of liver cancers and is the third leading cause of cancer related death in the world 1. In the past decades, the diagnosis and treatment methods for HCC have achieved great progress. However, the incidence of HCC continues to increase annually and the various treatments including radical treatments such as surgical resection, radiofrequency ablation and liver transplantation, do not markedly enhance overall survival in HCC patients 2. High rate of tumor recurrence and distant metastasis after hepatectomy is a major risk factor affecting its prognosis 3. Therefore, it is urgent to develop effective prognostic biomarkers and novel therapeutic targets for improving the survival of HCC patients.

The oncogenic transcription factor MYC protein plays a central role in regulating cell proliferation, cell adhesion, cellular metabolism, and the apoptosis 4. The MYC gene amplification and the over-expression of the MYC protein are frequently detected and significantly correlated with malignant potential and poor prognosis in human cancers including HCC 5, 6. The role of MYC in liver carcinogenesis also has been demonstrated *in vitro* and* in vivo* 7-9. Moreover, inactivation of MYC gene leads to complete tumor regression in the transgenic mouse model of MYC-driven HCC 10. In fact, the inhibitors targeting MYC have been tested in the clinical trials and have become a promising therapeutic option 11.

Long non-coding RNAs (LncRNAs) are a type of RNA molecules longer than 200 nt with little or no protein coding capacity. Increasing evidence has indicated that lncRNAs function as a miRNA sponge or protein scaffold to participate in various biological processes including cell metabolism 12, apoptosis 13, proliferation 14, 15, and stemness 16. Studies have demonstrated that lncRNAs are involved in regulating the proliferation and metastasis of HCC cells. For example, the lncRNA DANCR and UFC1 activate Wnt signaling via stabilizing CTNNB1 protein 17, 18; lncRNA Ptn-dt promotes the proliferation of HCC cells by interacting with HuR protein 19; linc-GALH promotes metastasis of HCC through controlling the methylation status of Gankyrin by adjusting the ubiquitination status of DNMT1 20. With the development of high-throughput microarray and RNA sequencing technology, an increasing number of lncRNAs have been identified to be associated with HCC, while the intrinsic functions and the underlying mechanisms of these lncRNAs have not yet been elucidated.

To systematically identify HCC-related lncRNAs, we performed lncRNA microarray analysis in 127 paired HCC and peritumor tissues. Here we identified an upregulated lncRNA RP11-134O21.1 (formally named lncCSMD1-1 in LNCipedia database will be here on refer to lncCSMD1-1 as lncCSMD1) in HCC. To date, there has been no reported study on the link between lncCSMD1 and cancers, and its function is also unclear. In this study, we report for the first time that high expression level of lncCSMD1 is correlated with metastasis and poor prognosis in patients with HCC. Mechanistically, lncCSMD1 specifically binds to MYC and inhibits its ubiquitination, leading to stabilization of MYC protein and activation of its downstream gene expression.

## Methods

### Patient samples

In this study, we first obtained 127 paired HCC and adjacent non-tumorous liver (ANL) tissues from patients as a Discovery Cohort. These patients were pathologically diagnosed with HCC and received radical surgery between December 2005 and November 2011 at Sun Yat-Sen University Cancer Center (SYSUCC), Guangzhou, China. None of the patients had received any treatment for cancer before radical resection. Samples from this cohort were detected with a custom lncRNA microarray. Another 260 HCC patients were collected as a Validation Cohort; these patients underwent radical surgery between February 2006 and September 2013 at the same cancer center, which were recruited according to the same criteria as in the Discovery Cohort. The Validation Cohort samples were detected with qRT-PCR to validate lncCSMD1 expression level and its clinical significance in HCC. In order to confirm the clinical significance of lncCSMD1 in different patient populations and geographical areas, we conducted a multicenter validation study: 94 HCC patients were recruited from a medical center in Jilin province, Northeast China, and 124 HCC patients from a medical center in Shanghai, East China, as External Validation Cohort 1 (Ext Valid Cohort 1) and External Validation Cohort 2 (Ext Valid Cohort 2), respectively. The recruitment criteria for the two cohorts was the same as in the Discovery Cohort. The samples from these two cohorts were also examined with qRT-PCR. The follow-up times for the Discovery, Validation, and External Validation 1 and 2 Cohorts ranged from 3~105 months (Mo) with median time 55.3 Mo, 2~77 Mo with median time 30.0 Mo, 3~56 Mo with median time 41.2 Mo, and 3-94 Mo with median time 45.7 Mo, respectively. Overall survival (OS) was defined as the time from the date of surgery to death or last follow up. Disease-free survival (DFS) was defined as the time from the date of surgery to relapse, distance metastasis, death or last follow up. All tissues were preserved in -80°C for subsequent analysis. The clinical characteristics of the four cohorts are listed in Table 1. This research was approved by the Research Ethics Committee of Sun Yat-Sen University Cancer Center and written informed consent was obtained from all the participants.

### Animal studies

Four- to six-week-old male BALB/c nude mice were purchased from Beijing Vital River Laboratory Animal Center (Beijing, China) and were used for this study with the approval of the Laboratory Animal Ethics Committee of Sun Yat-Sen University. The mice (5 for each group) were subcutaneously injected with 5×10^6^ Hep3B cells with stable expression of lncCSMD1 or control vector. Tumor size in mice was then measured using calipers every 5 days and tumor volume was calculated using a method previously described 21. All mice were euthanized 30 days post-treatment and their tumor tissues were resected, fixed with formalin and sectioned with a rotary microtome for hematoxylin-eosin (H&E) and Ki-67 immunohistochemical staining. To test the role of lncCSMD1 in tumor metastasis, another two groups of mice (5 for each group) were injected with 1.5×10^6^ Hep3B HCC cells stably expressing lncCSMD1 or control vector through the tail vein. Body weights of all mice were recorded and the experiment was terminated 90 days later. Their lungs were removed for pathological examination.

### Cell lines

The human HCC cell lines Hep3B, HepG2, MHCC97H, MHCC97L, SK-Hep-1 and a normal hepatocyte cell line LO2 were preserved in the State Key Laboratory of Oncology in South China, and cultured at 37°C in Dulbecco's modified Eagle's medium (Gibco, USA) plus with 10% fetal bovine serum (Invitrogen, USA), 100 units/ml penicillin, and 100 μg/ml streptomycin in a humidified atmosphere containing 5% CO_2_. SMMC7721 cells were maintained in RPMI1640 (Gibco, USA) supplemented with 10% FBS. All cell lines were authenticated by STR DNA profiling (Microread Diagnostics Co., Ltd, Guangzhou, China) and tested for *Mycoplasma* contamination by RT-PCR in our lab.

### Construction of stable cell lines

The full-length sequence of lncCSMD1 or short hairpin RNA (shRNA) against lncCSMD1 was amplified and cloned into the multiple cloning sites of pcDNA3.1, then subcloned into lentivirus to overexpress or knockdown lncCSMD1 by GenePharma (Shanghai, China), respectively. Following a 48-h period of infection with lentivirus plus 5 mg/ml Polybrene, stable cells with expression of lncCSMD1 or shRNA were selected with 4 μg/mL puromycin for 3 days. After selection, the cells were cultured with medium containing 2 μg/mL puromycin.

### RNA extraction and RT-qPCR

Total RNAs were extracted from HCC and adjacent non-tumor tissues or from cultured cells using TRIzol reagent (Invitrogen, CA, USA). Cytoplasmic and nuclear RNAs were extracted with PARIS kit (Life Technologies, USA). Complementary DNAs (cDNA) were obtained from reverse transcription of 1000 ng of total RNA using PrimeScript RT reagent Kit (Promega, Madison, WI, USA). Quantitative PCR was performed on the cDNA using GoTaq® qPCR Master Mix (Promega, Madison, WI, USA) according to the manufacturer's instructions on Roche LightCycler® 96 real-time PCR machine. Relative expression of lncRNA and relevant genes was normalized to GAPDH using 2^-ΔΔCT^ method. Primers used in this study are listed in Table S9.

### Cell proliferation, migration and invasion assays

Cell proliferation was assessed by CCK-8 Cell Counting Kit (Dojindo Laboratory, Kyushu, Japan) and colony formation assay. For CCK-8 assay, cells were seeded into 96-well plates at a density of 1000 cells per well and incubated for 7 days under 5 % CO_2_. After cells were treated with CCK-8 solution for 2 hours on the indicated days, the growth rate of cells was determined by absorbance at 450nm with SpectraMax M5 Multi-Mode Microplate Reader (Molecular Devices LLC, Sunnyvale, CA, USA). For colony formation assay, cells were seeded into 6-well plates (1000 cells per well) and cultured for 14 days, then fixed with methanol for 15 minutes and stained with 2% crystal violet solution for 1 hours. Images of Colonies were captured by ChemiDoc Imaging Systems (Bio-Rad, California, USA) and the number of colonies were counted by Image J software.

Cell migration and invasion assays were conducted with Transwell method (BD Biosciences, Lexington, UK), and Transwell method with matrigel on the bottom membrane (with 8-μm pore size) of the chamber, respectively. 1 × 10^5^ cells were seeded into the upper chamber with 300 μL serum-free medium, while in the lower chamber DMEM medium supplemented with 10% FBS was added. After 24 hours, migrated cells were fixed with methanol and stained with crystal violet (Weijia Biology Science and Technology Co., Guangzhou, China) and the number of migrated and invaded cells were counted using an Image J software. All the experiments were performed three times independently.

### Western blot analysis

For preparing proteins, cultured cells were lysed in radioimmune precipitation assay (RIPA) buffer plus phenylmethylsulfonyl fluoride (PMSF), protease and phosphatase inhibitors. Then the cell lysates were mixed with BCA solution and the protein concentration was determined by absorbance at 562 nm in SpectraMax M5 Multi-Mode Microplate Reader according to the instruction of the BCA protein assay kit (Beyotime, Haimen, China). Then 20 μg of protein extracts were subjected to western blot according to the standard procedure. The specific antibodies used in this study are as follows: β-Catenin (#8480), E-Cadherin (#3195), N-Cadherin (#13116), c-Myc (#9402), IgG (30000-0-AP), HA-Tag (#3724), FLAG (#14793), Ubiquitin (#3933), purchased from Cell Signaling Technology (Danvers, MA 01923, USA); C-MYC (10828-1-AP) and GAPDH (HRP-60004) purchased from Proteintech Group (Rosemont, IL 60018, USA). All antibodies were diluted to 1: 1000 in the blotting.

### Immunohistochemistry (IHC)

For Ki-67 immunohistochemical staining, paraffin-embedded tissues were cut into 4-μm-thick sections and endogenous peroxidase was eliminated using 3% hydrogen peroxide. The sections were boiled for 3 minutes in an electric pressure cooker to restore antigen and then blocked in 3% BSA for 1 hour. Next, the tissue slices were incubated with primary antibody against Ki-67 (#9449, 1:100, Cell Signaling Technology) at 4°C for 12 hours. After that, tissue sections were incubated with Horseradish Peroxidase (HRP)-conjugated secondary antibody and DAB solution. Finally, the sections were re-stained with hematoxylin. IHC score was quantified using ImageJ software (Maryland, USA).

### Immunofluorescence (IF) analysis

IF analysis was conducted on HCC slices for examining the expression and subcellular location of MYC protein. Briefly, HCC tissues were embedded in paraffin and sectioned to 4 μm slices. Then the slices underwent antigen retrieval via a pressure steam chamber for 3 minutes. After being blocked in 10% FBS for 1 hour, the sections were incubated with primary antibody against C-MYC (10828-1-AP, 1:100, Proteintech) at 4°C for 14 hours. Next, the secondary antibody conjugated with Alexa Fluor 488 Dye (Invitrogen, CA, USA) was used to label the primary antibody for 1 hour. Then the sections were co-stained with 4′ 6-diamidino-2-phenylindole (DAPI) (Beyotime, Shanghai, China). Photographs were captured and analyzed using the LSM 880 confocal laser scanning microscope (ZEISS, Jena, German).

### Gene expression array and GSEA analysis

Total RNAs were extracted from cultured HCC cells stably overexpressing lncCSMD1 or control vector using TRIZOL Reagent (Invitrogen, CA, USA) according to manufacturer's instruction. The quantity and quality of total RNAs were measured using Agilent Bioanalyzer 2100 (Agilent technologies, Santa Clara, USA). Then RNeasy mini kit and RNase-Free DNase Set (both sets from QIAGEN, GmBH, Germany) were used to purify total RNAs. Next, the purified total RNAs were subjected to Affymetrix PrimeView Human Gene Expression Array conducted by Bohao Biotech Company, Shanghai, China.

Differentially expressed genes (DEGs) between the HCC cells with overexpressing lncCSMD1 and control vector were identified with SAM program and then analyzed by gene set enrichment analysis (GSEA) software (Broad Institute, San Diego, USA) to find gene sets enriched by lncCSMD1 overexpression. The settings for GSEA analysis were made according to the instructions of the software.

### RNA Fluorescence *in situ* hybridization (RNA-FISH)

RNA-FISH analysis in HCC cells and primary HCC liver tissues were carried out according to the standard protocol. The RNA probes conjugated with Cy3 at 5'-end for detection of lncCSMD1 or control RNA were purchased from Ribo Biotechnology Co. Ltd (Guangzhou, China). The specific probe sequence of lncCSMD1 was as follows: 5'-AAGACATTGATGCCCATGAAAGACCAAAAGTAACAAAACAACAACAGAAAACCAGGGG-3'. Cell nuclei were stained with DAPI (Beyotime, Shanghai, China). Fluorescence signal was observed and photographed with a LSM 880 confocal laser scanning microscope (ZEISS, Jena, German). The relative fluorescence intensity was quantified using ImageJ software (Maryland, USA).

### Immunoprecipitation (IP)

Cultured cells were lysed in Pierce™ IP Lysis Buffer (Invitrogen, CA, USA) supplemented with PMSF, protease inhibitor and phosphatase inhibitor. Protein concentrations were measured with a BCA protein assay kit (Beyotime, Haimen, China) and subsequently 1 mg lysates were incubated with unconjugated primary antibody against Ubiquitin-HA-tag (#3724, 1:50, Cell Signaling Technology) or MYC-Flag-tag (#3724, 1:50, Cell Signaling Technology) at 4°C overnight followed by 1 h incubation with Protein G Agarose (Sigma, Burlington, USA) for separating the antibody. The precipitated protein complexes were washed using IP lysis buffer for 4 times before being separated on SDS-PAGE and/or immunobloting by the indicated antibodies.

### RNA pull-down assay

RNA pull-down assays were carried out as previously described 22. Briefly, the full-length sense and its antisense RNA sequences of lncCSMD1 were transcribed *in vitro* using MAXIscript T7 kit (Invitrogen, CA, USA) and then purified with EasyPure® RNA Kit (Transgene Biotech, Beijing, China). The purified sense and its antisense lncRNAs were then labeled with desthiobiotin at 3'-End using Pierce™ RNA 3' End Desthiobiotinylation Kit (Invitrogen, CA, USA) and mixed with nuclear proteins, which were extracted from HCC cells stably overexpressing lncCSMD1 or control vector using ProteinExt® Mammalian Nuclear and Cytoplasmic Protein Extraction Kit (Transgene Biotech, Beijing, China). Next, the lncRNA-protein complexes were purified with Pierce™ Magnetic RNA-Protein Pull-Down Kit (Invitrogen, CA, USA) and then separated on polyacrylamide gel electrophoresis (PAGE). Finally, the separated proteins were stained with Fast Silver Stain Kit (Beyotime, Shanghai, China) followed by mass spectrum to identify the specific proteins binding to lncRNA; immunobloting was then used to validate the interaction.

### RNA immunoprecipitation (RIP) assay

RIP assay was performed using BersinBio^TM^ RNA Immunoprecipitation (RIP) Kit (BersinBio, Guangzhou, China) according to the manufacturer's instructions. The total RNA extracted from HCC cells was mixed and precipitated with antibody against MYC (#9402, 1:50, Cell Signaling Technology) or control antibody (IgG). The precipitated RNA was detected by quantitative RT-PCR to evaluate the level of lncCSMD1. The primers for RT-PCR are presented in Table S9.

### Statistical analysis

R language (version 3.53, Missouri, USA) packages (Gmisc and boot) were used to analyze the relationship between lncCSMD1 expression and clinical features of HCC patients and *p* value was calculated by Chi squared (χ2) test. Cox's proportional hazards regression model and Kaplan-Meier Method and log-rank test were conducted on OS and DFS in GraphPad Prism 7 software (San Diego, CA, USA). Two-tailed Student's t-test was adopted to evaluate the differences between groups. *P* < 0.05 was considered statistically significant.

## Results

### lncCSMD1 is upregulated and positively correlated with disease progression and poor prognosis in multicenter HCC patients

In this study, we first collected 127 HCC patients as a Discovery Cohort from SYSUCC, Guangzhou, China. To screen differentially expressed lncRNAs in HCC, we analyzed the lncRNA profile in the 127 pairs of HCC and ANL samples from the Discovery Cohort using a custom microarray containing 2412 lncRNA probes. There are 218 lncRNAs identified to be differentially expressed between HCC and ANL tissues, of which 85 lncRNAs are upregulated and 133 downregulated. Next, univariate Cox proportional hazard regression analysis was performed on the 218 differentially expressed lncRNAs, and 14 deregulated lncRNAs (6 upregulated and 8 downregulated) were found to be associated with the prognosis of HCC patients (data will be published separately).

**Figure 1:**
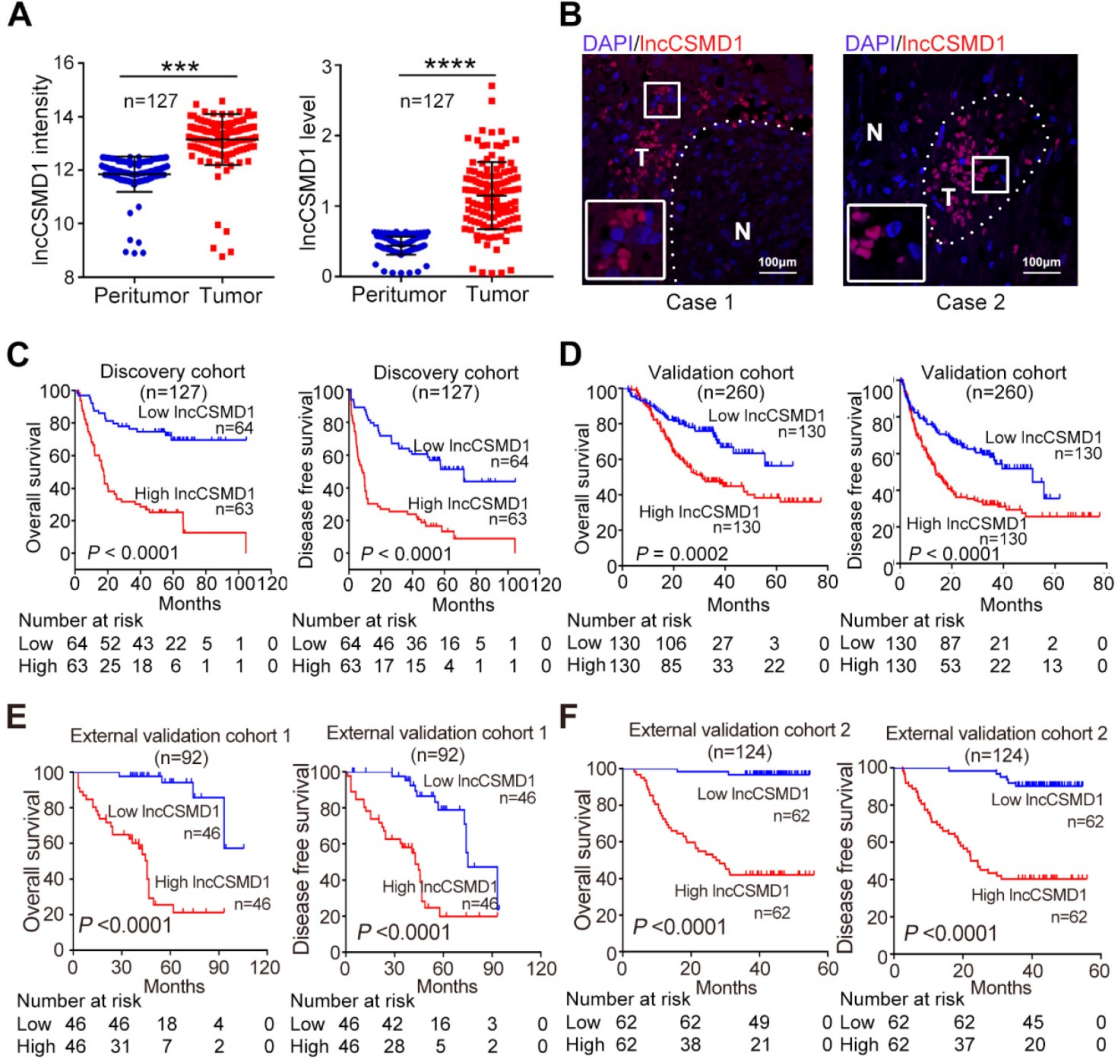
**lncCSMD1 expression is increased and positively correlated with poor prognosis in HCC patients**. **(A)** Relative lncCSMD1 expression level was measured in 127 paired HCC and peritumor liver tissues (Discovery Cohort) with a custom lncRNA microarray (**left**) and in another 127 paired HCC and peritumor liver tissues (randomly selected from the Validation Cohort) with qRT-PCR (**right**). **(B)** lncCSMD1 was mainly expressed in HCC tissues (**T**), but rarely in peritumor liver tissues (**N**), as shown by RNA fluorescence in situ hybridization.** (C)** In the Discovery Cohort, 127 HCC patients were divided into high or low expression group according to the median value of lncCSMD1 expression detected by custom lncRNA microarray. Kaplan-Meier analysis revealed that HCC patients with high lncCSMD1 expression level have significantly worse overall survival (OS, left) and disease-free survival (DFS, right) than those with low lncCSMD1 level.** (D-F)** The HCC patients in the Validation Cohort (**D**), External Validation Cohort 1 (**E**) and 2 (**F**) were divided into high or low expression group according to the median value of lncCSMD1 level detected by qRT-PCR. Kaplan-Meier analysis of these Validation Cohorts verified the results obtained in the Discovery Cohort.

Among these 14 deregulated lncRNAs, lncCSMD1 is most significantly overexpressed in HCC tissues (**Figure 1A left**). To validate this result, we applied RNA FISH in two HCC samples and found higher signals of lncCSMD1 in HCC cells compared with ANL cells (**Figure 1B**). Then, the HCC patients were divided into high- and low-expression groups based on the median level of lncCSMD1 in HCC tissues. Correlation analysis on the lncCSMD1 expression levels and clinical characteristics shows that lncCSMD1 expression is positively related with HBsAg positive, cirrhosis, tumor number, TNM stage, and metastasis (**Table S1**). Next, Survival analysis shows that high lncCSMD1 expression is significantly correlated with poor OS and DFS of HCC patients (**Figure 1C**, both *p*<0.001). Cox regression analysis indicates that lncCSMD1 is a significant prognostic factor for OS and DFS in HCC patients (**Table 2**). These results indicate that lncCSMD1 is a potential biomarker for survival prediction in HCC patients.

To verify this potential prognostic factor, we recruited another 260 HCC patients as a Validation Cohort. We detected lncCSMD1 expression with qRT-PCR in 260 HCC tissues and 127 paired ANL samples randomly selected from the 260 HCC cases. The result exhibits that LncCSMD1 is a 2.62-fold upregulated in the 127 HCCs as compared with that in the paired ANLs (**Figure 1A right**, *p*<0.001), and higher lncCSMD1 expression in HCC tissues than in the paired ANL tissues can be detected in 93.7% (119/127) of the cases (**Figure S1**). Then 260 HCC patients were separated into low- and high-expression groups using the median lncCSMD1 expression level as the cut-off value. Correlation analysis shows that high lncCSMD1 expression is only associated with HBsAg positive (**Table S2**). Cox regression analysis indicates that lncCSMD1 is a significant predictor for OS and DFS (**Table 2**), and HCC patients with high lncCSMD1 expression have much worse OS and DFS (**Figure 1D**, both *p*<0.001) than those with low lncCSMD1, which confirm the results obtained by microarray analysis in the Discovery Cohort.

To further confirm the clinical role of lncCSMD1 in patients from different geographic areas, we recruited 94 HCC patients from Jilin province, Northeast China, as the first External Validation Cohort (Ext Valid Cohort 1), and 124 HCC patients from Shanghai, East China, as the second External Validation Cohort (Ext Valid Cohort 2). We also examined lncCSMD1 expression by qRT-PCR in the two cohorts. These patients were then classified into high- and low-expression groups based on their lncCSMD1 expression level in HCC tissues. Higher lncCSMD1 expression is significantly correlated with pathology grade, HBV DNA, tumor embolus, tumor capsule, tumor number, tumor size, clinical stage in Ext Valid Cohort 1 (**Table S3**) and/or Ext Valid Cohort 2 (**Table S4**). Cox regression analysis demonstrates that lncCSMD1 is also a significant prognostic factor for OS and DFS (**Table 3**) in the two cohorts, and high lncCSMD1 expression is also significantly associated with OS and DFS in the Ext Valid Cohort 1 (**Figure 1E**) and/or Ext Valid Cohort 2 (**Figure 1F**) (both *p*<0.001). Finally, we performed multivariate Cox analysis to identify whether lncCSMD1 was an independent prognostic factor in HCC patients. The result shows that lncCSMD1 is a significantly independent prognostic factor for OS in Validation Cohort, External Validation Cohort 1 and 2 (all *p*<0.05, **Table S5 and S6**) but not for the Discovery Cohort (*p*=0.18, **Table S5**). We surmised that the lack of statistical significance may be attributed to a small number size and/or larger heterogeneity of patients in the Discovery Cohort. Therefore, we combined the four cohorts into one set (total 603 patients) and repeated the Cox analysis. Expectedly, univariate and multivariate Cox analyses demonstrate that lncCSMD1 is a significant prognostic factor for OS and DFS in all HCC patients (all *p*<0.001, **Table S7 and S8**), indicating that lncCSMD1 is an independent survival predictor for HCC patient. Altogether, lncCSMD1 not only correlated with tumor progression but may also be a novel and powerful survival predictor for HCC patients in the multicenter study, implying that lncCSMD1 plays a critical role in the development and progression of HCC.

### lncCSMD1 promotes proliferation, migration and invasion of HCC cells *in vitro*

**Figure 2:**
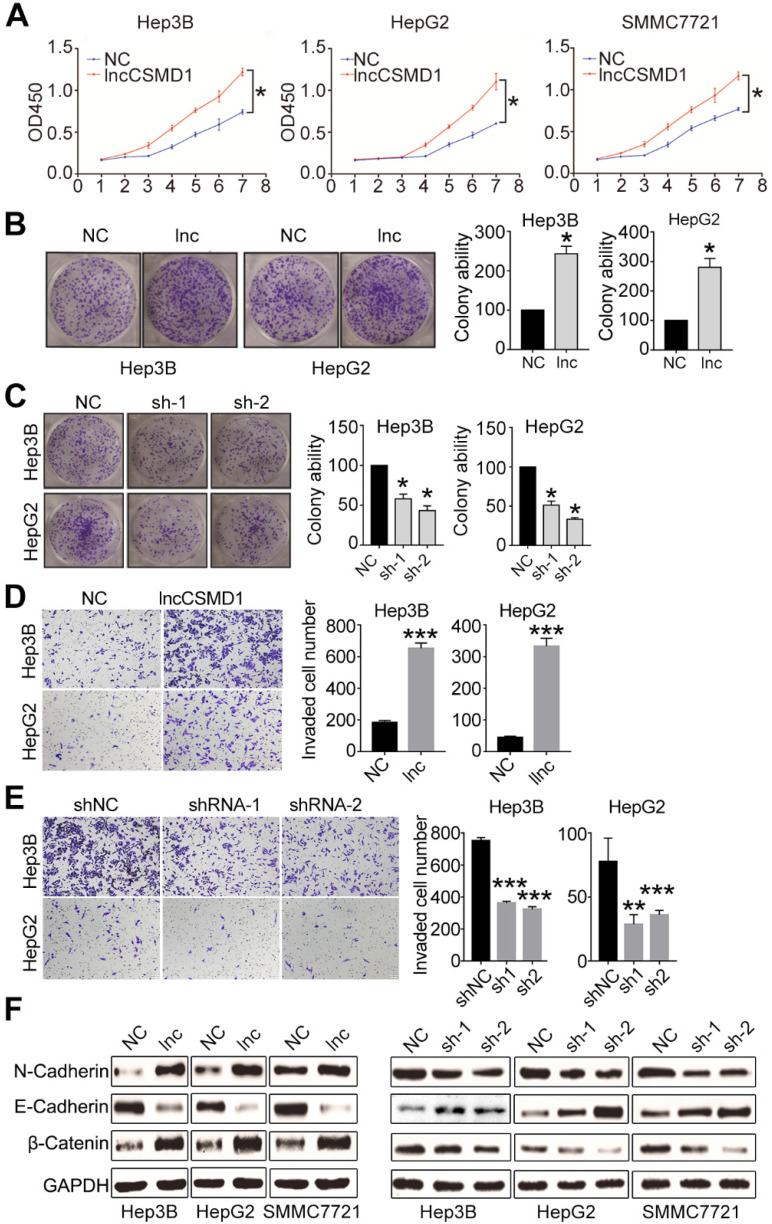
**Ectopic expression of lncCSMD1 promotes proliferation, colony formation and invasion of HCC cells *in vitro*. (A)** The ectopic expression of lncCSMD1 markedly promotes cell proliferation in Hep3B, HepG2 and SMMC7721 HCC cells, which were determined by CCK-8 assay. **(B)** lncCSMD1 overexpression significantly enhances colony formation in Hep3B and HepG2 HCC cells compared with the control cells. Data represent the mean ± SD of triplicate experiments and was analyzed by Student t test. * means p <0.05. (also applies to the following). **(C)** Downregulated lncCSMD1 notably inhibits colony formation in Hep3B and HepG2 HCC cells compared with the control cells. (**D**) In transwell assay, lncCSMD1 expression strikingly raises the cell invasion in Hep3B and HepG2 cells compared with the control cells. (**E**) In transwell assay, downregulated lncCSMD1 by shRNA markedly reduces the cell invasion in Hep3B and HepG2 cells compared with the control cells. (**F**) Overexpression of lncCSMD1 causes the expression of mesenchymal proteins (N-cadherin and β-catenin) and reduces the expression of epithelial protein (E-cadherin) in Hep3B, HepG2 and SMMC7721 cells, as shown by western blot.

To confirm the important role of lncCSMD1 in hepatocarcinogenesis, we first investigated whether it can affect the proliferation of HCC cells. To this end, we checked the transcript level of lncCSMD1 in six HCC cell lines (Hep3B, HepG2, SMMC7721, MHCC97H, MHCC97L and Sk-Hep-1) and a normal hepatocyte cell line (LO2) with qRT-PCR, and found that lncCSMD1 was significantly increased in all six HCC cell lines when compared with LO2 cells (**Figure S2A**), which is concordant with that in HCC tissues. To elucidate the biological functions of lncCSMD1 in HCC, we performed gain- or loss-of-function studies by stably overexpressing or knocking down lncCSMD1 in three HCC cell lines (Hep3B, HepG2 and SMMC7721). The relative expression of lncCSMD1 in the 3 HCC cells transfected with lentivirus containing lncCSMD1 cDNA or shRNA or control vector was confirmed by qRT-PCR (**Figure S2B-C**). We then did CCK8 and colony formation assays to analyze the effect of the increased lncCSMD1 on the HCC cells, and found that the 3 HCC cells with lncCSMD1 overexpression had significantly higher proliferation rate (*p*<0.05, **Figure 2A**) and higher colony number (**Figure 2B and Figure S2D**) than those in the control HCC cells. Immunohistochemistry for PCNA in Hep3B and HepG2 also exhibits that lncCSMD1 enhances the proliferation index of HCC cells (**Figure S2E**). When lncCSMD1 was knocked down by shRNA in Hep3B, HepG2 and SMMC7721 HCC cells, the colony number was remarkably reduced (**Figure 2C and Figure S2F**). These results indicate that lncCSMD1 can promote oncogenic growth of HCC cells.

Next, we investigated whether the lncCSMD1 could impact the migration and invasion of HCC cells. Transwell assay was conducted in the 3 HCC cells stably overexpressing or less-expressing lncCSMD1. The result showed that more migrated cells were observed in the membrane of wells inoculated with HCC cells stably overexpressing lncCSMD1 (**Figure S3A-B**), and wound scratch assay also indicated that the Hep3B cells with lncCSMD1 overexpression migrated faster than the control cells (**Figure S3C**). When lncCSMD1 was knocked down by shRNA in Hep3B, HepG2 and SMMC7721 cells, HCC cells migrated much less in the transwell membrane when compared with the control HCC cells (**Figure S3D**). We then explored the effect of lncCSMD1 on the invasion of HCC cells. Transwell with matrigel assay revealed that lncCSMD1 overexpression could promote the invasion of Hep3B and HepG2 (**Figure 2D**), while down-regulation of lncCSMD1 restrained the invasion of HCC cells (**Figure 2E**). These results demonstrate that lncCSMD1 can enhance the migration and invasion ability of HCC cells.

Epithelial-mesenchymal transition (EMT) was reported to be involved in migration, invasion and metastasis of various cancer cells 23. To explore the role of lncCSMD1 in the EMT process, we analyzed EMT markers by western blotting in the 3 HCC cell lines with stable overexpression or downregulation of lncCSMD1. The result indicated that lncCSMD1 enhanced the protein level of mesenchymal markers N-cadherin and β-catenin but decreased the epithelial marker E-cadherin in Hep3B, HepG2 and SMMC7721 cells (**Figure 2F left**). The opposite results were obtained from HCC cells with stably downregulation of lncCSMD1 (**Figure 2F right**). Collectively, the above results suggest that lncCSMD1 induces migration and invasion via enhancing EMT in HCC cells.

### lncCSMD1 promotes tumor growth and distant metastasis of HCC cells *in vivo*

**Figure 3:**
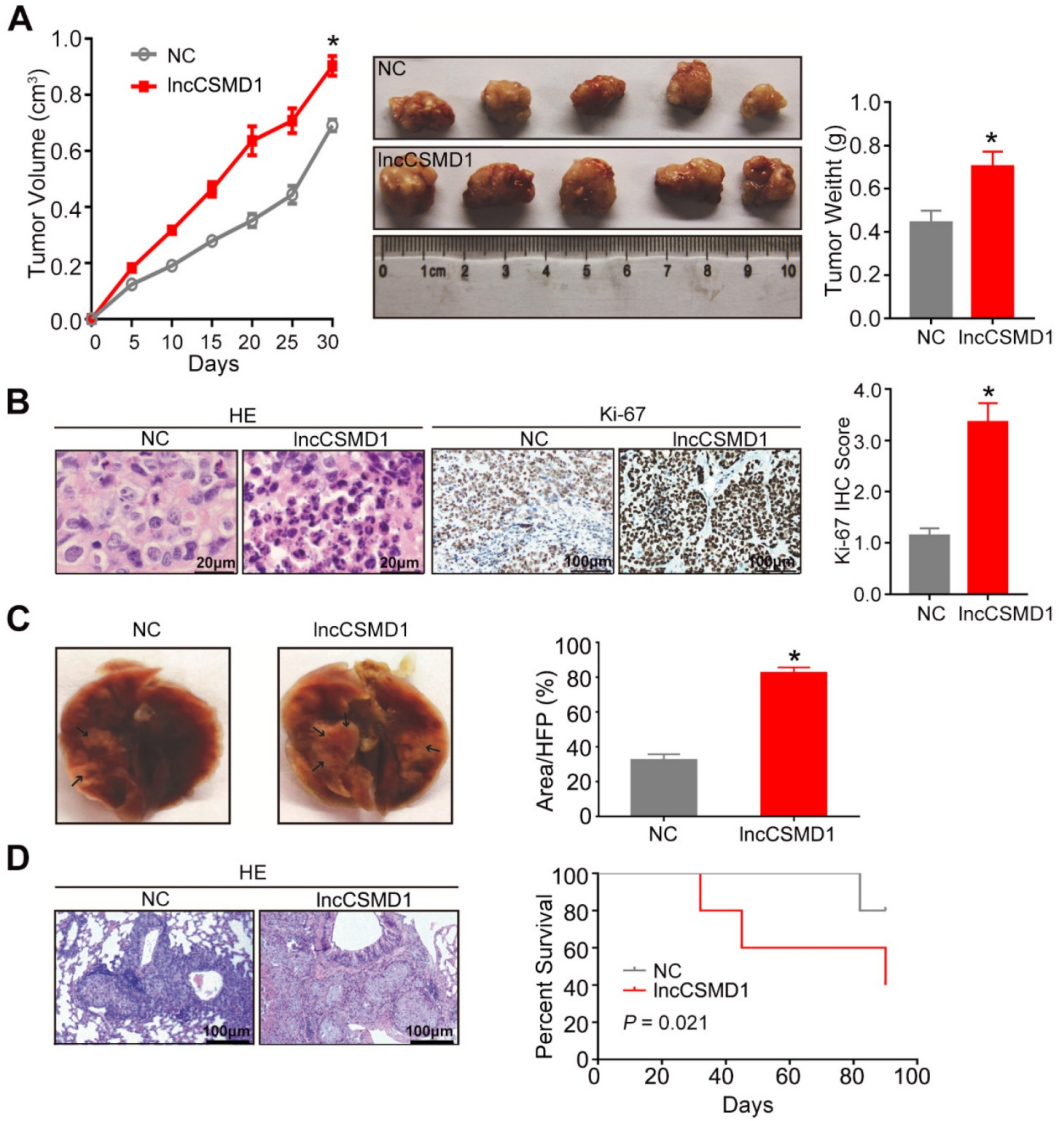
** LncCSMD1 promotes growth and metastasis of xenograft tumors derived from Hep3B HCC cells.** (**A**) In the tumor growth curve, the tumors grow faster in the mice subcutaneously inoculated with Hep3B cells overexpressing lncCSMD1 than in mice with the HCC cells carrying a control vector (**left**); Images of xenograft tumors derived from Hep3B cells stably overexpressing lncCSMD1 or carrying a control vector (**middle**); the weights of xenograft tumors in the two groups were compared with histogram (**right**), in which data were analyzed by sample-paired t test; *P <0.05 (the followings are the same). (**B**) Representative images of HE staining (**left**) and Ki67 IHC staining (**middle**) displayed the pathological configuration and proliferation capacity of HCC xenograft cells; IHC scores for Ki67 were compared in the two groups with histogram (**right**). (**C**) Representative images of nude mouse lungs with metastatic foci derived from tail-vein injected Hep3B cells stably overexpressing lncCSMD1 or carrying a control vector (n=5 mice/each group); the metastatic foci ratios (foci to the observed lung area) were compared in the two groups with histogram. (**D**) Representative images of metastatic foci stained with HE in the lung tissue sections (**left**); survivals mice tail-vein injected with Hep3B cells stably expressing lncCSMD1 or a control vector were analyzed with Kaplan-Meier curve (**right**).

To further investigate the biological role of lncCSMD1 *in vivo*, we built a xenograft model in nude mice by subcutaneous injection of 5x10^6^ Hep3B cells stably overexpressing lncCSMD1 or an empty vector, and evaluated the xenograft tumor volume in mice every 5 days using a method previously described. On the 30th day after Hep3B cells were injected subcutaneously, all mice were euthanized, and xenograft tumors were resected, weighed, fixed in 10% buffered formalin and sectioned in a rotary microtome. The tumors from HCC cells stably overexpressing lncCSMD1 grew faster than those from the control HCC cells (**Figure 3A left**), and the tumor weights were significantly increased in the lncCSMD1 overexpression group compared with the control group (**Figure 3A middle and right**). Under a microscope, the xenograft tumors were confirmed to be HCCs in H&E stained slices by experienced pathologist (**Figure 3B left**), and lncCSMD1 was corroborated to be highly expressed in the xenograft tumor from HCC cells overexpressing lncCSMD1 (**Figure S3E**). Furthermore, Ki-67 IHC scores were much higher in the tumors with lncCSMD1 overexpression (**Figure 3B middle and right**).

To validate the results that lncCSMD1 promoted migration, invasion and EMT of HCC cells *in vitro*, we constructed a lung metastasis mouse model by tail-vein injection of Hep3B cells with overexpression of lncCSMD1 or control vector. At the seventh week after injection, all the mice were euthanized, and whole lungs were harvested for computing the number of metastatic foci (**Figure 3C**). Compared with the control group, the number of metastatic foci in the lung was dramatically increased in the lncCSMD1 overexpression group by counting of metastatic foci under a microscope (**Figure 3D left**). More important, survival analysis suggests that mice injected with HCC cells expressing lncCSMD1 have shorter survival time compared with the control mice (**Figure 3D right**). Altogether, lncCSMD1 facilitates tumor growth and metastasis of HCC cells *in vivo*.

### lncCSMD1 directly interacts with protein MYC

**Figure 4:**
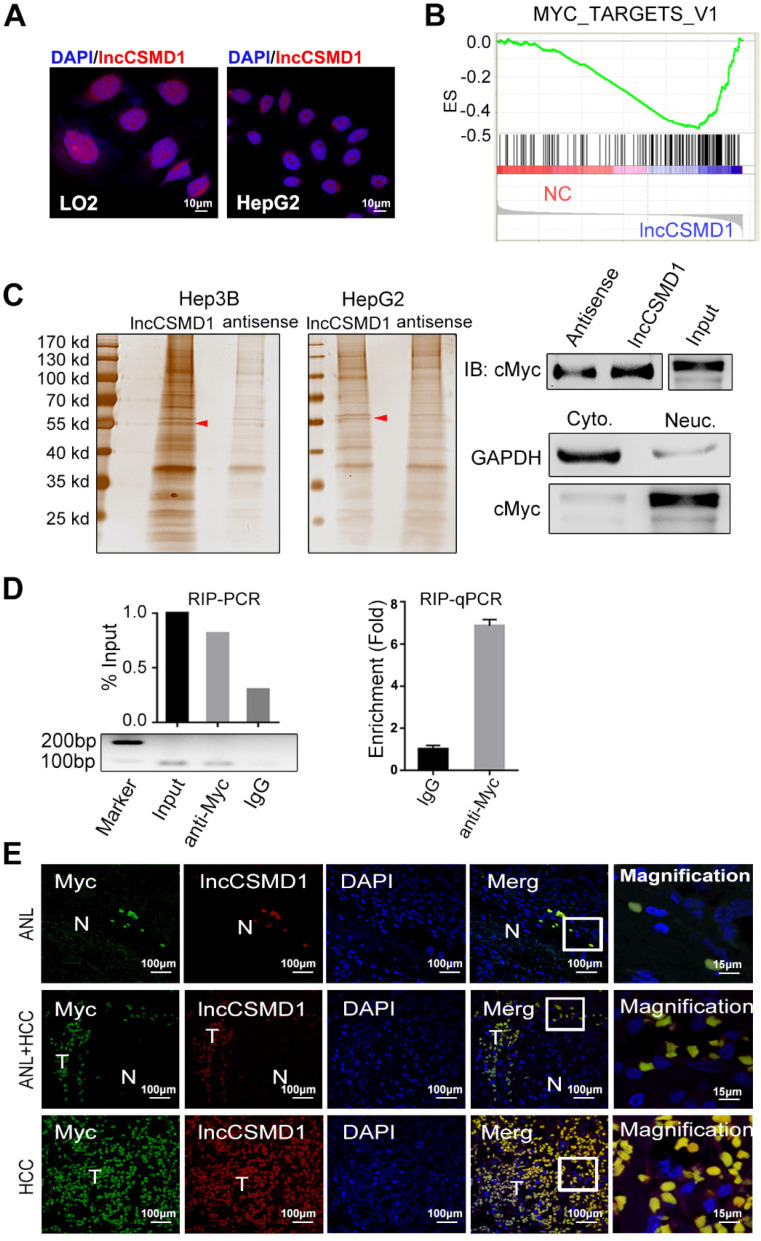
** LncCSMD1 directly interacts with MYC protein.** (**A**) LncCSMD1 is located in the nucleus of HepG2 and LO2 cells, as shown by RNA-FISH assay; (**B**) Gene set enrichment analysis (GSEA) for gene expression array data from Hep3B cells stably expressing lncCSMD1 and a control vector indicates a significant association between lncCSMD1 and MYC target gene signature. (**C**) Silver staining images of PAGE gels, in which lncCSMD1/proteins complexes from RNA pull down experiments on nucleus proteins of HCC cells stably expressing lncCSMD1 were separated; the arrows show the specific protein bands in pull down complexes by lncCSMD1 sense sequence when compared with antisense sequence (**left**); Immunoblot analysis verified the interaction between MYC and lncCSMD1 sense sequence (**right**). (**D**) LncCSMD1 PCR products amplified from RNA immunoprecipitation (RIP) complexes are separated on Agarose gel (**lower left**); the lncCSMD1 PCR products are quantified and compared with histogram (** upper left**); quantitative PCR was used to compare the lncCSMD1 amount that binds to MYC protein in the RIP experiment (**right**). (**E**) MYC protein is overlapped with lncCSMD1 RNA in the nucleus of HCC and peritumor liver cells, as displayed by Immunofluorescence (for MYC protein) coupled with fluorescence in situ hybridization (for lncCSMD1). T: HCC tissue; N: liver tissue.

To uncover the molecular mechanism underlying the tumor-promoting effect of lncCSMD1, we first performed RNA FISH in HCC tissues and cell lines to delineate the subcellular distribution of lncCSMD1 because the potential functions of lncRNAs are generally associated with their subcellular localization patterns. In HCC tissues, the fluorescence was mainly located in the DAPI stained nuclei of HCC cells (**Figure 1B**). This was confirmed in HepG2 and LO2 cells (**Figure 4A**). The RNAs extracted from cytoplasm and nucleus respectively, was measured with qRT-PCR assay, and the result also indicates that lncCSMD1 is mainly located in the nucleus (**Figure S3F**). These results suggest that lncCSMD1 may be involved in regulation of gene transcription. To explore the effect of lncCSMD1 on gene expression, we compared the gene expression profiles between the cells with lncCSMD1 overexpression and control vector using gene microarray, and then conducted gene set enrichment analysis (GSEA) on the gene profiles. GSEA revealed that MYC target V1 and V2 gene sets were enriched in cells with lncCSMD1 overexpression (**Figure 4B and Figure S4A-B**), implying that lncCSMD1 might be involved in the activation of MYC pathway.

To assess how lncCSMD1 affects MYC pathway, we first performed RNA pull down assay using biotin labeled lncCSMD1 sense and antisense probes mixed with nucleus proteins from Hep3B and HepG2 cells. As shown in polyacrylamide gel, a specific band of 55KD was emerged in the pull-down complex by lncCSMD1 sense probe when compared with the complex by antisense probe (**Figure 4C left, red arrow and Figure S5**). The specific band was removed for mass spectrometry (MS) assay, and MYC protein was identified in this band by MS. Then we verified the identified MYC protein in this band with western blot analysis (**Figure 4C right**). To further substantiate this finding, we carried out RNA immunoprecipitation (RIP) assay on nucleus proteins using antibody against MYC and non-specific IgG. Consistently, enhanced enrichment of lncCSMD1 RNA was detected with RT-PCR in MYC-RNA complex precipitated by MYC antibody in contrast to that by IgG antibody (**Figure 4D left**). On the other hand, if the two molecules interact, they must be in the same subcellular location. Therefore, we carried out RNA FISH assay to detect lncCSMD1 and IF assay to locate MYC protein in HCC tissues. As expected, we observed that lncCSMD1 RNA completely overlapped with MYC protein within the nuclei in both HCC and ANL tissues (**Figure 4E**). Altogether, these results demonstrated lncCSMD1 could directly bind to MYC protein in the nucleus of HCC cells.

### lncCSMD1 enhances MYC protein by inhibiting ubiquitin-proteasome pathway

**Figure 5:**
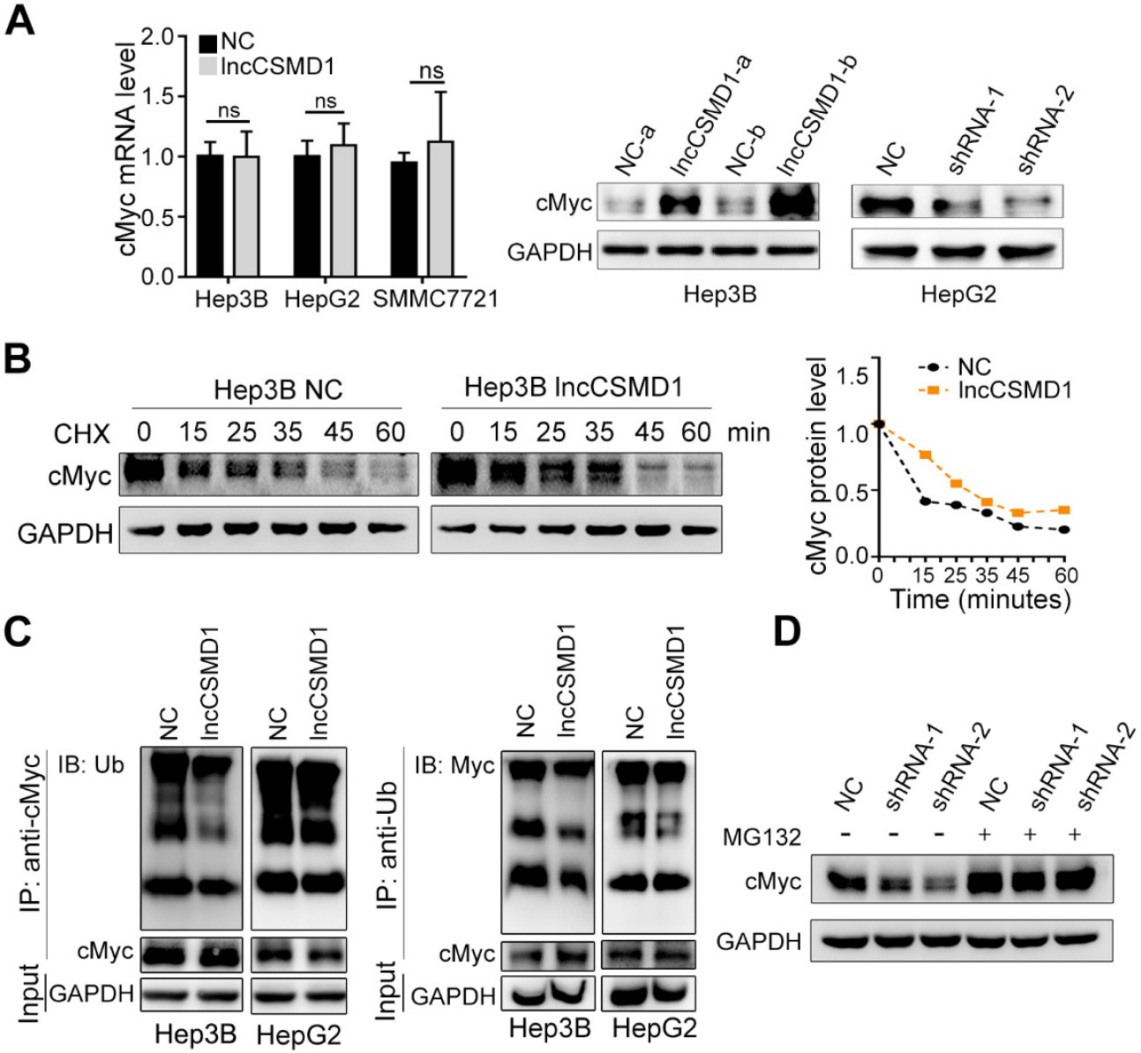
** LncCSMD1 stabilized MYC protein by inhibiting its ubiquitination**. (**A**) MYC mRNA expression level in Hep3B cells stably expressing lncCSMD1 is not significantly higher than that in the control cells, as exhibited by qRT-PCR (**left**); however, MYC protein level is much higher in Hep3B cells stable overexpression of lncCSMD1 than in control cells (**middle**) or obviously lower in HepG2 cells with lncCSMD1 downregulation than in control cells (**right**). (**B**) Hep3B cells were treated with cycloheximide (CHX; 20 μg/ml) for the indicated times, and the half-life of MYC protein in HCC cells with overexpressing lncCSMD1 is prolonged when compared with that in control cells, as revealed by immunobloting (**left**); the curves were used to compare the half-life times of MYC protein in Hep3B cells with overexpression of lncCSMD1 or vector (**right**). (**C**) HCC cells overexpressing lncCSMD1 or vector were treated with MG132 (5 μM) for 24 h, and then cell lysates were immunoprecipitated with MYC antibody and the precipitated complexes were subjected to western blot with ubiquitin antibody (**left**); or the cell lysates were immunoprecipitated with ubiquitin antibody and the precipitated complexes were subjected to western blot with MYC antibody (**right**). The two experiments are aimed to detect the ubiquitination status of MYC protein. (**D**) MYC protein in Hep3B cells with downregulation of lncCSMD1 by shRNA is reduced compared with that in negative control cells, while after MG132 (20 μM) treatment for 24 h, MYC protein is restored to the same level as that in control cells, as shown by western blot assay.

MYC protein is a transcription factor that regulates a diverse array of gene expressions and plays an important role in human carcinogenesis 24. As MYC has a short half-life, its activity level can be elevated by raising its mRNA level or prolonging protein half-life when it functions as an oncogenic protein in tumorigenesis. Thus, we first wanted to know whether lncCSMD1 could impact the mRNA level of MYC. With qRT-PCR assay, we found that there was no significant alteration of the level of MYC mRNA in Hep3B, HepG2 and SMMC7721 HCC cells with overexpression of lncCSMD1 compared with the corresponding control cells (**Figure 5A left**), indicating that lncCSMD1 does not regulate MYC at the transcriptional level. Based on the above result of lncCSMD1 directly binding to MYC protein, we surmised that lncCSMD1 could affect the protein level of MYC. As shown by western blot, MYC protein level was substantially increased when lncCSMD1 was overexpressed in Hep3B cells (**Figure 5A middle**), and markedly diminished when lncCSMD1 was knocked down by shRNAs in HepG2 cells (**Figure 5A right**), suggesting that lncCSMD1 can elevate MYC protein level at the post-transcriptional level.

In general, the elevated level of protein can be caused by increasing its synthesis or prolonging its half-life time. Because no elevation of MYC mRNA was observed in HCC cells with lncCSMD1 overexpression, we evaluated the half-life period of MYC protein in Hep3B cells. After HCC cells with and without stably overexpressing lncCSMD1 were treated with protein synthesis inhibitor cycloheximide (CHX), the half-life of MYC protein was observed to be longer in the cells with stably overexpressing lncCSMD1 than that in the control cells (**Figure 5B**), implying that lncCSMD1 can maintain the stability of MYC protein. Since ubiquitination is a main degradation way for MYC protein 25, we further assessed the ubiquitination level of MYC protein when lncCSMD1 was overexpressed in Hep3B and HepG2 cells compared with control cells by co-immunoprecipitation (Co-IP). First, nucleus proteins were co-precipitated by MYC antibody. Then the precipitated proteins were detected with ubiquitin antibody on western blot. As expected, we found that the ubiquitinated MYC proteins precipitated from the cells with lncCSMD1 overexpression was notably reduced compared with that from the control cells (**Figure 5C left**). Correspondently, the ubiquitinated MYC protein precipitated from the same cells by ubiquitin antibody also decreased compared with that from the control cells (**Figure 5C right**). These results demonstrate that lncCSMD1 can reduce the ubiquitination of MYC protein. To confirm whether lncCSMD1 inhibits the degradation of MYC protein via ubiquitin-proteasome pathway, HepG2 cells with lncCSMD1 knockdown were treated with proteasome inhibitor MG132. The result shows that endogenous MYC protein in lncCSMD1 knockdown HepG2 cells was reduced, while the MYC protein level in the same HepG2 cells was increased to the same level as that in the negative control cells after MG132 treatment (**Figure 5D**). These results demonstrate that lncCSMD1 enhances MYC protein through suppression of degradation of MYC via inhibiting ubiquitin-proteasome pathway.

### lncCSMD1 promotes HCC progression via activating MYC signaling pathway

**Figure 6:**
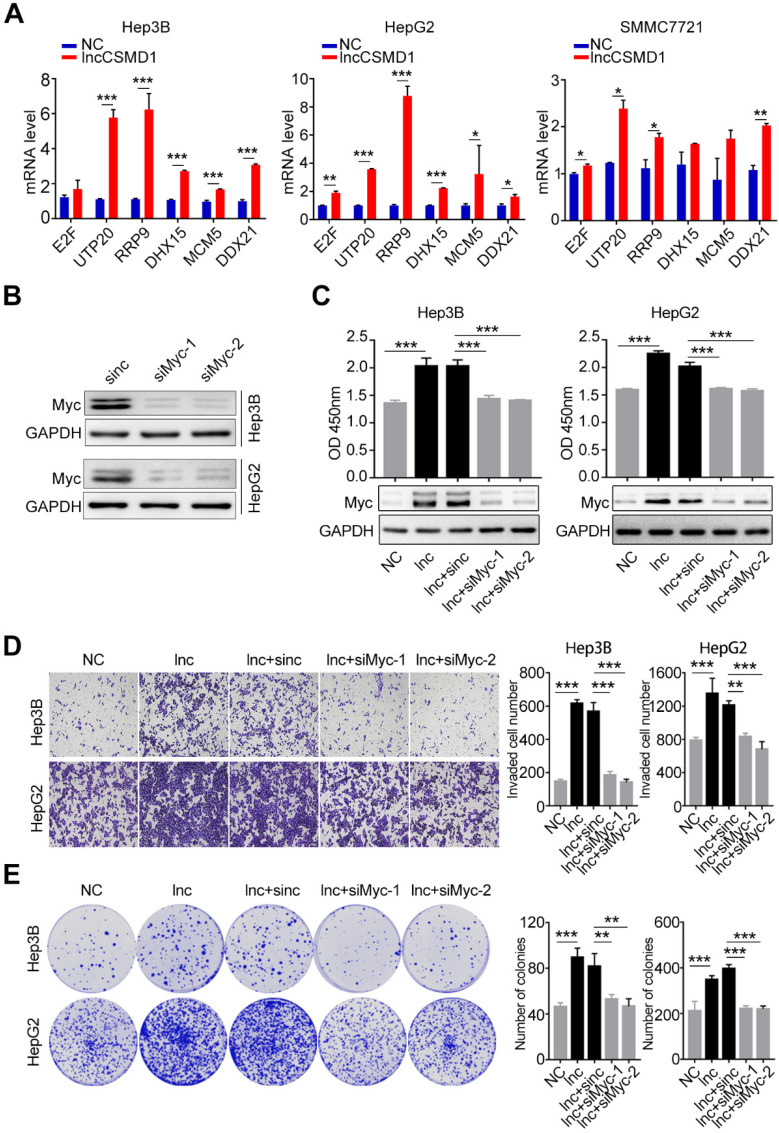
**MYC protein plays a key role in lncCSMD1-induced malignant phenotypes of HCC cells.** (**A**) MYC target genes in Hep3B, HepG2 and SMMC7721 cells with overexpression of lncCSMD1 are expressed higher than those in the control cells, as shown by qRT-PCR. (**B**) MYC protein expression is reduced by siRNA against MYC in Hep3B and HepG2 cells, as shown by Western Blot. (**C**) MYC protein in Hep3B and HepG2 cells with lncCSMD1 overexpression is elevated, as displayed by western blot (**lower panels**) and promotes cell proliferation as shown by CCK-8 assay (**upper panels**) compared with that in control cells; when the HCC cells were treated with siMYC again, MYC protein was reduced and leads to decrease in cell proliferation. (**D, E**) The same treatments as in the above (C) were conducted in the same Hep3B and HepG2 cells, and these cells were determined with transwell assay (D) and colony formation assay (E), in which overexpressed lncCSMD1 promotes the invasion and colony formation of the HCC cells and the downregulated MYC by siRNA inhibits colony formation and invasion of the HCC cells.

In recent years, intensive studies have focused on the function and mechanism of lncRNAs in cancer and found that lncRNAs generally function by serving as a scaffold to affect protein modification. MYC is a critical transcription factor that regulates transcription of downstream target genes, leading to enhanced proliferation and metastasis of diverse cancers. As described above, we found that lncCSMD1 could enhance MYC protein level and functioned as an oncogene to facilitate proliferation, migration, invasion and EMT of HCC cells. Thus, we investigated whether lncCSMD1 promoted HCC progression by raising MYC pathway activity. To this end, we examined the expression of downstream target genes transcribed by MYC with qRT-PCR. In line with the result of gene expression array as described above, the mRNA levels of MYC target genes were notably increased in Hep3B, HepG2 and SMMC7721 cells with lncCSMD1 overexpression (**Figure 6A and Figure S6**), implying that lncCSMD1 promoted hepatocarcinogenesis by activating MYC signaling pathway. Further protein-protein interaction (PPI) network analysis on gene expression profiles reveals that these genes are mainly involved in RNA processing and cell cycle (**Figure S4C**).

**Figure 7:**
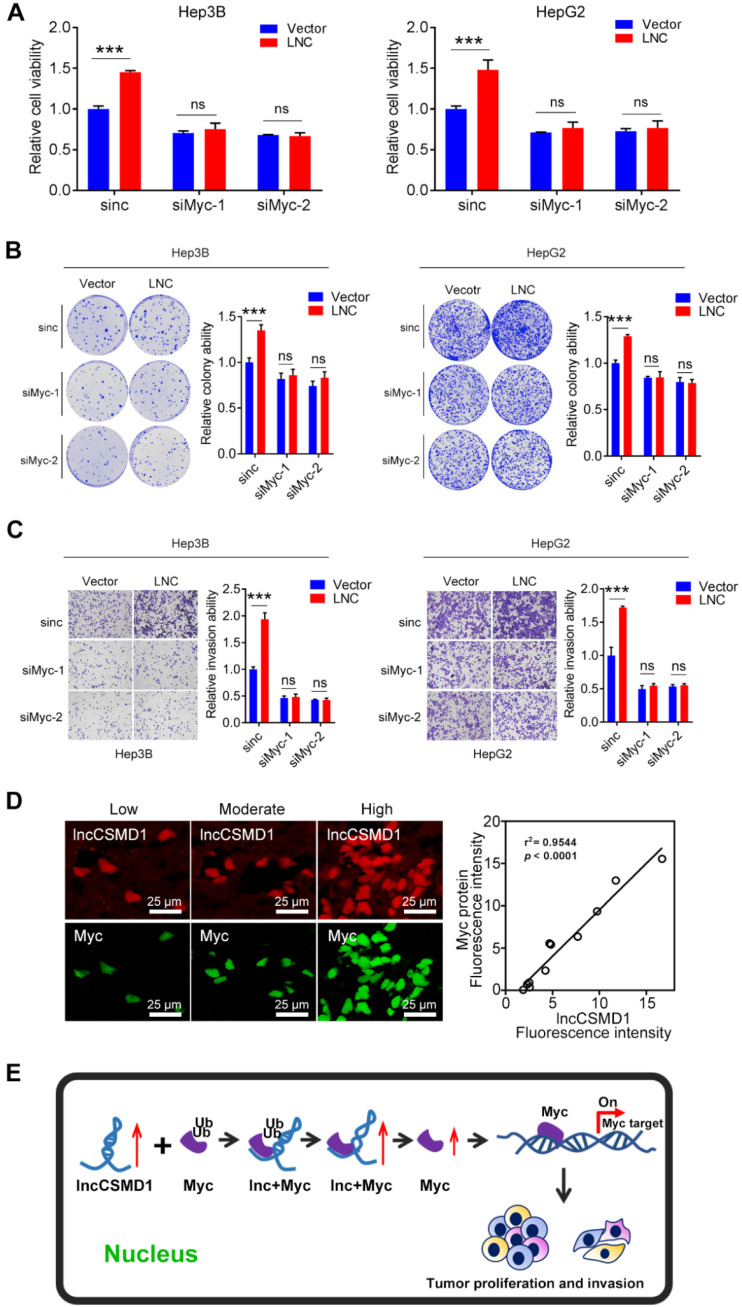
**LncCSMD1 failed to induce malignant phenotypes of HCC cells under knock-down of MYC.** (**A-C**) In Hep3B and HepG2 cells, MYC was knocked down by siRNA against MYC followed by transient overexpression of lncCSMD1, and then CCK8 (A), colony formation (B) and invasion assay (C) were carried out to evaluate the effect of lncCSMD1 overexpression on the proliferation, colony formation and invasion ability of these HCC cells, showing that lncCSMD1 overexpression can promote proliferation, colony formation and invasion of the control HCC cells, but has not any significant effect on the biological phenotypes of the HCC cells with downregulation of MYC compared with those of the HCC cells with only MYC knockdown. (**D**) The expressions of lncCSMD1 RNA and MYC protein in 11 primary HCC tissues were investigated by FISH and immunofluorescence, respectively, and the representative images are presented (**left**); In Pearson correlation analysis, lncCSMD1 RNA has a highly positive relationship with MYC protein in HCC tissues (11 cases) (**right**). (**E**) The work model of lncCSMD-induced oncogenic phenotypes of HCC cells via activating MYC signaling.

To further confirm that MYC plays a central role in lncCSMD1-induced oncogenic functions in HCC, we conducted a rescue experiment on HepG2 and Hep3B cells overexpressing lncCSMD1. First, we treated these two parental HCC cells with MYC-specific siRNA and observed reduced MYC protein levels in the two cells (**Figure 6B**), suggesting that the two siRNAs against MYC work well in the two HCC cells. Then we performed the rescue experiment on the two cells overexpressing lncCSMD1 with the siRNAs against MYC. As expected, western blot assay showed that the upregulated MYC protein level induced by lncCSMD1 was abolished by the siRNA in the two HCC cells (**Figure 6C lower panel**); more important, the increased cell proliferation (**Figure 6C upper panel**), invasion (**Figure 6D**), colony formation (**Figure 6E**) and migration (**Figure S7**) by lncCSMD1 overexpression were also completely attenuated by the siRNA against MYC compared with the control cells, indicating that lncCSMD1-induced oncogenic phenotypes are mediated by MYC protein. To further confirm the key role of MYC protein in lncCSMD1-induced oncogenic phenotypes, we performed the second rescue experiment on Hep3B and HepG2 cells. The two cells were first treated with Myc-siRNAs followed by transient overexpression or downregulation of lncCSMD1, respectively, and then CCK8, colony formation and invasion assay were performed on these cells. Not unexpected, the results indicate that the lncCSMD1 overexpression cannot rescue the inhibition of cell proliferation (**Figure 7A**), colony formation (**Figure 7B**) and invasion (**Figure 7C**) of the HCC cells induced by knockdown of MYC compared with the control cells, and lncCSMD1 downregulation also cannot further inhibit cell proliferation, colony formation and invasion of HCC cells with MYC knockdown (**Figure S8**) compared with the control cells. Again, these results demonstrate that MYC protein mediates lncCSMD1-induced oncogenic phenotypes. In addition, immunofluorescence coupled with RNA FISH assay was performed to localize and quantify the expression of lncCSMD1 and MYC protein in the primary HCC tissues (n=11), and the result indicates that both lncCSMD1 RNA and MYC protein are located in the nuclei of HCC cells and have a close and positive relationship (**Figure 7D and Figure S9**). Altogether, lncCSMD1 promotes hepatocarcinogenesis via boosting MYC signaling pathway activity.

## Discussion

In this study, we reveal for the first time that lncCSMD1 is notably upregulated in HCC tissues in a Discovery cohort (127 pairs of HCC and ANL samples) and we validate it in another 127 pairs of HCC and ANL tissues (random selection from the Validation Cohort) with qRT-PCR method; more important, we demonstrate that high lncCSMD1 expression is significantly associated with poor OS and DFS in HCC patients with microarray analysis in a Discovery Cohort (127 HCC cases), and validate this finding with RT-PCR method in a Validation Cohort (260 cases) and two External Validation Cohorts (92 and 124 cases, respectively), suggesting that lncCSMD1 is a novel and reproducible prognostic biomarker for HCC patients and play an important role in HCC progression. To our best knowledge, there is no report on the biological role, clinical significance and mechanism of lncCSMD1 in cancers. Recently, one study shows that lncCSMD1 expression is elevated in a subtype of B cell precursor acute lymphoblastic leukemia (BCP-ALL) 26, which is concordant with our result. In addition, the overexpressed lncCSMD1 is correlated with its promoter hypomethylation in BCP-ALL, which may also be the reason for overexpression of lncCSMD1 in HCC. However, that study did not explore the role, clinical significance and mechanism of lncCSMD1 in BCP-ALL.

LncCSMD1 is located on chromosome 8 short arm (8p23), and its transcript length is 569 bases. In this study, as described above, functional studies demonstrated that overexpression of lncCSMD1 induced a remarkable proliferation, migration, invasion and EMT *in vitro*, and tumor growth and metastasis *in vivo*, providing the evidence that lncCSMD1 functions as an "oncogene" and promotes the development and progression of HCC.

In the recent decade, lncRNAs have been reported to play critical roles in tumorigenesis and progression of various cancers 27, 28. Mechanistically, lncRNA is capable of regulating various cell signalings in carcinogenesis, such as Wnt/β-catenin 29, NOTCH 30, and MAPK 31. To understand the molecular mechanisms underlying the oncogenic role of lncCSMD1 in HCC, we first explored gene expression profile and cellular signalings regulated by lncCSMD1 with commercial microarray, GSEA and RT-PCR analysis, and found that lncCSMD1 enhances MYC protein expression and activates MYC signaling without affecting its mRNA expression at the transcriptional level, implying that MYC and its signaling play an important role in lncCSMD1 mediated hepatocarcinogenesis.

As an important oncogenic transcription factor, MYC has been identified as a powerful driver gene and a central regulator of malignant transformation in human hepatocarcinogenesis 32, 33. In animal models, invasive HCC could be induced by MYC activation 34. In general, the function of a gene or protein is determined by its subcellular location. For example, cytoplasmic lncRNA mainly plays oncogenic or tumor suppressive roles by sponging miRNA 35, 36, and nucleic lncRNA usually functions as a scaffold for diverse nucleic proteins 37, 38. In this study, we observed that lncCSMD1 RNA and MYC protein are co-localized in the nuclei of HCC cells and tissues, and lncCSMD1 is directly bound to MYC protein, suggesting that lncCSMD1 acts as a MYC scaffold to regulate MYC downstream targets.

Studies have shown that lncRNA plays important roles in regulating gene expression at three levels: gene transcription, RNA stability and protein stability/activity 39, 40. Since lncCSMD1 does not impact MYC mRNA level, we reason that the lncCSMD1 maintains the stability of MYC protein via binding to the protein. As expected, our results confirmed that lncCSMD1 specifically protects MYC from degradation via inhibiting the ubiquitin-proteasome pathway, which stabilizes MYC protein, and simultaneously enhances MYC signaling pathway in HCC cells. Next, we conducted rescue experiment to demonstrate that the repression of MYC expression by specific siRNA can markedly abolish the enhanced proliferation, migration and invasion ability induced by lncCSMD1 overexpression in HCC cells, further confirming that lncCSMD1 promotes progression of HCC via enhancing MYC signaling. In addition, MYC signaling is also reported to regulate metabolism 41-43 and stemness 44 of cancer cells. Whether the oncogenic functions of lncCSMD1 in HCC are mediated by affecting metabolism and stemness induced by MYC in HCC remains to be elucidated.

In conclusion, in this study, we identified a novel lncRNA termed lncCSMD1 that is upregulated in HCC tissues and positively correlated with metastasis and poor prognosis of HCC patients; in cell and animal models, we validated the oncogenic role of lncCSMD1 in HCC; mechanistically, we demonstrated that lncCSMD1 specifically binds MYC protein and maintains its stability by reducing the ubiquitination of MYC, leading to activation of MYC downstream signaling pathway in HCC cells. Collectively, our findings provide the evidence that lncCSMD1 promotes hepatocarcinogenesis by stabilizing MYC protein and may be considered as a new potential biomarker for prognostic prediction and target for HCC therapy in the future.

## Supplementary Material

Supplementary figures and tables.

## Figures and Tables

**Table 1:** Clinical characteristics of HCC patients in the four cohorts

Characteristics	Discovery	Validation	Ext valid	Ext valid
	Cohort	Cohort	Cohort 1	Cohort 2
	N=127	N=260	N=92	N=124
**Age (years)**				
≥50	66 (52.0%)	134 (51.5%)	15 (16.3%)	9 (7.3%)
<50	61 (48.0%)	126 (48.5%)	77 (83.7%)	115 (92.7%)
**Gender**				
Female	16 (12.6%)	134 (51.5%)	66 (71.7%)	76 (61.3%)
Male	111 (87.4%)	126 (48.5%)	26 (28.3%)	48 (38.7%)
**AFP (μg/L)**				
>400	76 (59.8%)	171 (65.8%)	57 (62.0%)	75 (60.5%)
≤400	51 (40.2%)	89 (34.2%)	35 (38.0%)	49 (39.5%)
**HBsAg**				
Negative	14 (11.0%)	27 (10.4%)	23 (29.9%)	47 (37.9%)
Positive	113 (89.0%)	233 (89.6%)	54 (70.1%)	77 (62.1%)
**HBV DNA**				
Negative	41 (32.3%)	73 (28.1%)	29 (35.4%)	36 (31.6%)
Positive	86 (67.7%)	187 (71.9%)	53 (64.6%)	78 (68.4%)
**Pathology Grade**				
I	3 (2.4%)	17 (6.5%)	18 (20.7%)	3 (4.8%)
II	91 (71.7%)	164 (63.1%)	46 (52.9%)	13 (20.6%)
III	33 (26.0%)	79 (30.4%)	23 (26.4%)	47 (74.6%)
**Cirrhosis**				
NO	53 (41.7%)	108 (41.5%)	14 (15.2%)	14 (11.3%)
Yes	74 (58.3%)	152 (58.5%)	78 (84.8%)	110 (88.7%)
**Main Size**				
<5cm	56 (44.1%)	103 (39.6%)	48 (52.2%)	55 (44.4%)
≥5cm	71 (55.9%)	157 (60.4%)	44 (47.8%)	69 (55.6%)
**Tumor Number**				
1	87 (68.5%)	172 (66.2%)	70 (76.1%)	41 (33.1%)
≥2	40 (31.5%)	88 (33.8%)	22 (23.9%)	83 (66.9%)
**Tumor capsule**				
No	43 (33.9%)	91 (35.0%)	70 (76.1%)	56 (45.2%)
Yes	84 (66.1%)	169 (65.0%)	22 (23.9%)	68 (54.8%)
**Cancer Embolus**				
No	102 (80.3%)	195 (75.0%)	23 (25.0%)	64 (51.6%)
Yes	25 (19.7%)	65 (25.0%)	69 (75.0%)	60 (48.4%)
**TNM stage**				
I	68 (53.5%)	111 (42.7%)	43 (46.7%)	13 (10.5%)
II	28 (22.0%)	77 (29.6%)	10 (10.9%)	42 (33.9%)
III	31 (24.4%)	72 (27.7%)	39 (42.4%)	69 (55.6%)

**Table 2:** Univariate Cox analysis of lncCSMD1 expression and clinical characteristics associated with survival in the Discovery and Validation Cohorts

	Discovery Cohort		Validation Cohort	
Characteristics	N=127		N=260	
	HR (95% CI)	P value	HR (95% CI)	P value
**Overall survival**				
lncCSMD1	3.30 (1.90-5.60)	<0.001	2.10 (1.40-3.10)	<0.001
Age	0.81 (0.50-1.30)	0.390	0.80 (0.55-1.20)	0.770
Gender	0.94 (0.48-1.80)	0.850	0.93 (0.55-1.60)	0.230
AFP	1.70 (1.10-2.80)	0.029	1.60 (1.10-2.30)	0.300
HBsAg	1.80 (0.73-4.50)	0.200	1.50 (0.71-3.00)	0.230
HBV DNA	1.30 (0.77-2.20)	0.310	1.10 (0.70-1.60)	0.830
Pathology Grade	1.60 (0.99-2.60)	0.054	1.40 (1.00-2.00)	<0.001
Cirrhosis	2.20 (1.30-3.70)	0.004	0.95 (0.65-1.40)	<0.001
Main Size	2.80 (1.60-4.70)	<0.001	2.00 (1.30-3.00)	0.730
Tumor Number	4.80 (2.90-7.90)	<0.001	2.00 (1.40-2.90)	<0.001
Tumor capsule	0.55 (0.34-0.90)	0.017	0.93 (0.63-1.40)	<0.001
Cancer Embolus	3.80 (2.30-6.40)	<0.001	2.40 (1.60-3.60)	0.044
TNM stage	3.00 (2.30-4.10)	<0.001	1.80 (1.40-2.20)	<0.001
Metastasis	2.30 (1.40-3.90)	0.002	3.60 (2.30-5.50)	<0.001
Relapse	2.00 (1.30-3.30)	0.004	2.30 (1.60-3.40)	<0.001
**Disease free survival**				
lncCSMD1	2.60 (1.70-4.10)	<0.001	2.10 (1.40-3.10)	<0.001
Age	0.78 (0.51-1.20)	0.260	0.80 (0.55-1.20)	0.230
Gender	1.10 (0.56-2.10)	0.820	0.93 (0.55-1.60)	0.780
AFP	1.80 (1.20-2.70)	0.010	1.60 (1.10-2.30)	0.013
HBsAg	1.80 (0.83-3.90)	0.130	1.50 (0.71-3.00)	0.300
HBV DNA	1.30 (0.84-2.20)	0.220	1.00 (0.69-1.60)	0.830
Pathology Grade	1.30 (0.84-2.00)	0.250	1.40 (1.00-2.00)	0.044
Cirrhosis	2.00 (1.30-3.20)	0.003	0.95 (0.65-1.40)	0.770
Main Size	2.20 (1.40-3.40)	<0.001	2.00 (1.30-3.00)	<0.001
Tumor Number	3.80 (2.40-5.90)	<0.001	2.00 (1.40-2.90)	<0.001
Tumor capsule	0.58 (0.37-0.90)	0.015	0.93 (0.63-1.40)	0.730
Cancer Embolus	2.70 (1.60-4.40)	<0.001	2.40 (1.60-3.60)	<0.001
TNM stage	2.50 (1.90-3.30)	<0.001	1.80 (1.40-2.20)	<0.001
Metastasis	3.00 (1.80-4.90)	<0.001	3.60 (2.30-5.50)	<0.001
Relapse	4.50 (2.90-7.00)	<0.001	2.30 (1.60-3.40)	<0.001

**Table 3:** Univariate Cox analysis of lncCSMD1 expression and clinical characteristics associated with survival in the two External Validation Cohorts

	Ext valid Cohort 1		Ext valid Cohort 2	
Characteristics	N=92		N=124	
	HR (95% CI)	P value	HR (95% CI)	P value
**Overall survival**				
lncCSMD1	11.0 (3.70-30.0)	<0.001	27.0 (6.4-110.0)	<0.001
Age	3.70 (0.87-15.0)	0.076	0.62 (0.22-1.70)	0.370
Gender	1.20 (0.58-2.60)	0.600	1.10 (0.55-2.00)	0.870
AFP	1.30 (0.67-2.70)	0.410	1.80 (0.95-3.40)	0.072
Pathology Grade	4.00 (2.60-6.30)	<0.001	7.80 (3.20-19.0)	<0.001
Cirrhosis	6.20 (0.84-45.0)	0.073	1.50 (0.47-4.90)	0.490
Main Size	2.10 (1.00-4.30)	0.038	2.10 (1.00-4.10)	0.038
Tumor Number	5.00 (2.50-10.0)	<0.001	24.00 (3.3-170)	0.002
Tumor capsule	3.30 (1.70-6.60)	<0.001	0.71 (0.37-1.30)	0.280
Cancer Embolus	0.24 (0.12-0.47)	<0.001	0.09 (0.03-0.25)	<0.001
TNM stage	1.50 (1.00-2.10)	0.039	2.10 (1.20-3.90)	0.011
**Disease free survival**				
lncCSMD1	3.50 (1.80-6.80)	<0.001	9.70 (4.10-23.0)	<0.001
Age	1.70 (0.67-4.30)	0.270	0.67 (0.24-1.90)	0.450
Gender	1.40 (0.75-2.70)	0.280	0.96 (0.52-1.80)	0.890
AFP	1.40 (0.75-2.60)	0.290	1.70 (0.95-3.10)	0.074
Pathology Grade	2.80 (1.90-4.10)	<0.001	6.60 (3.00-14.0)	<0.001
Cirrhosis	2.50 (0.76-8.00)	0.130	0.77 (0.33-1.80)	0.550
Main Size	1.50 (0.83-2.80)	0.170	1.60 (0.87-3.00)	0.130
Tumor Number	3.10 (1.60-5.80)	<0.001	14.0 (3.40-57.0)	<0.001
Tumor capsule	2.80 (1.50-5.20)	0.001	0.81 (0.44-1.50)	0.480
Cancer Embolus	0.31 (0.17-0.56)	<0.001	0.09 (0.04-0.24)	<0.001
TNM stage	1.20 (0.85-1.60)	0.330	1.70 (1.00-2.80)	0.041

## Abbreviations

ANLs: adjacent non-tumor livers
ANOVA: analysis of variance
Co-IP: co-immunoprecipitation
DDX21: DExD-box helicase 21
DFS: Disease-Free Survival
DHX15: DEAH-box helicase 15
E2F: transcription factor 1
EMT: epithelial-mesenchymal transition
FISH: fluorescence *in situ* hybridization
GAPDH: glyceraldehyde 3-phosphate dehydrogenase
GSEA: gene sets enrichment analysis
HCC: hepatocellular carcinoma
IHC: immunohistochemistry
lncRNA: long non-coding RNAs
MCM5: minichromosome maintenance complex component 5
miRNA: microRNA
mRNA: messenger RNA
MYC proto-oncogene: bHLH transcription factor
NC: negative control
OS: Overall Survival
PCR: polymerase chain reaction
PPI: protein-protein interaction
RIP: RNA immunoprecipitation
RRP9: ribosomal RNA processing 9 [U3 small nucleolar RNA binding protein]
shRNA: short hairpin RNA
siRNA: small interfering RNA
UTP20: UTP20 small subunit processome component
