# Supplementary material for: LncRNA CSMD1-1 promotes the progression of Hepatocellular Carcinoma by activating MYC signaling
Source: Theranostics. 2020 Jun 12;10(17):7527–44. doi: 10.7150/thno.45989 (PMC7359090; doi:10.7150/thno.45989)
Supplement: Supplementary file 1 — Supplementary figures and tables. [file thnov10p7527s1.pdf]

Supplemental Materials

LncRNA CSMD1 promotes proliferation and invasion of hepatocellular carcinoma by activating MYC signaling

Ji Liu[*1], Rui Xu[*2], Shi-Juan Mai[*1], Yu-Shui Ma[3], Mei-Yin Zhang[1], Ping-Sheng Cao[3], Nuo-Qing Weng[1], Rui-Qi Wang[1], Di Cao[1], Wei Wei[4], Rong-Ping Guo[4], Yao-Jun Zhang[4], Li Xu[4], Min-Shan Chen[4], Hui-Zhong Zhang[5], Long Huang[6], Da Fu[#3], and Hui-Yun Wang[#1].

1. Supplemental Figures: 9 figures.

2. Supplemental Tables: 9 tables.

1. SUPPLEMENTAL FIGURES

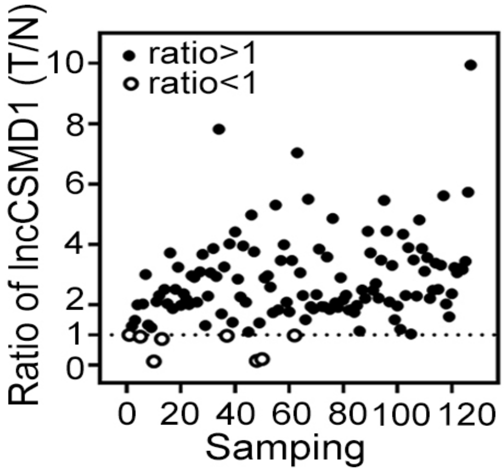

Figure S1. LncCSMD1 is upregulated in HCC tissues. lncCSMD1 expression level was detected with qRT-PCR in 127 paired HCC and adjacent non-tumor liver tissues randomly selected from the Validation Cohort. LncCSMD1 expression ratio (tumor to peritumor liver) > 1 was observed in 93.7% (119/127) of HCC tissues.

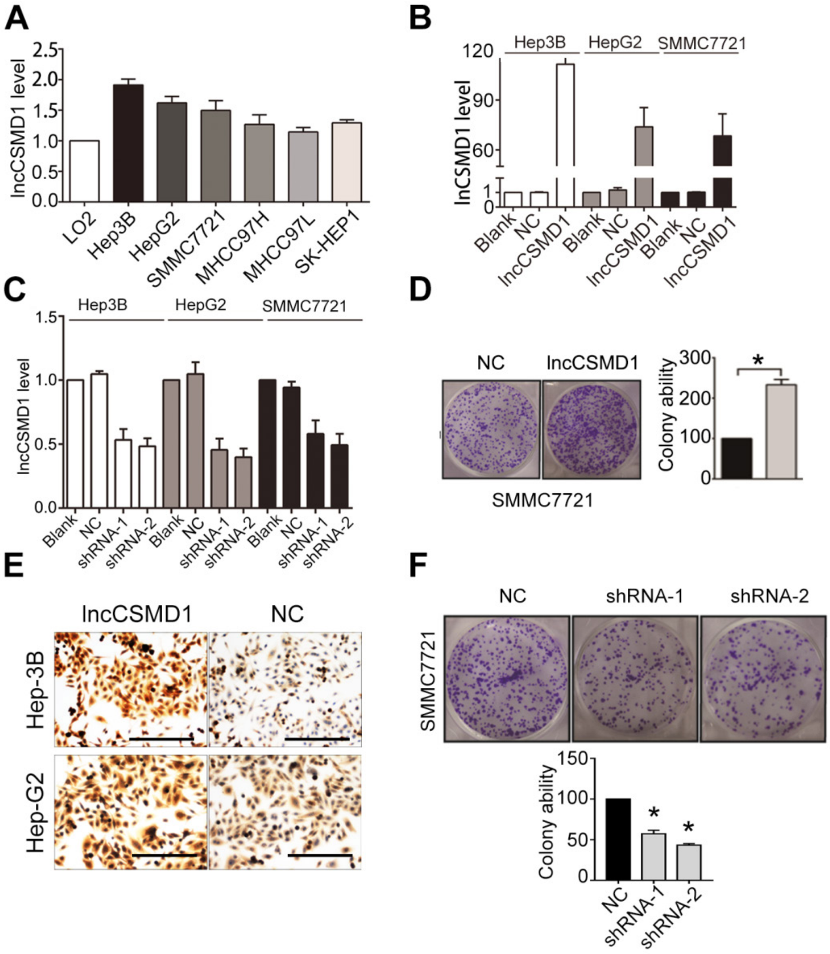

Figure S2. lncCSMD1 overexpression enhances growth of HCC cells. (A) qRT-PCR analysis shows that lncCSMD1 is upregulated in 6 HCC cells when compared with that in a normal liver cell. (**B**) lncCSMD1 is stably overexpressed in Hep3B, HepG2 and SMMC7721 cells transfected with lentivirus containing lncCSMD1 compared with the control cells, as shown by qRT-PCR. **(C)** LncCSDM1 expression was downregulated by shRNA against lncCSMD1 in Hep3B, HepG2 and SMMC7721 cells, as determined by qRT-PCR. (**D**) Ectopic expression of lncCSMD1 facilitates colony formation of SMMC7721 cells compared with the control cells. (**E**) High expression of PCNA protein in the Hep3B and HepG2 cells with stable overexpression of lncCSMD1 was detected with IHC compared with the control cells, suggesting that HCC cells with overexpression of lncCSMD1 have higher proliferation index. (**F**) HCC cells with lncCSMD1 downregulation exhibit fewer Colonies than the control HCC cells. Data represent the mean ± SD of triplicate experiments and was analyzed by Student t test. $*p < 0.05$.

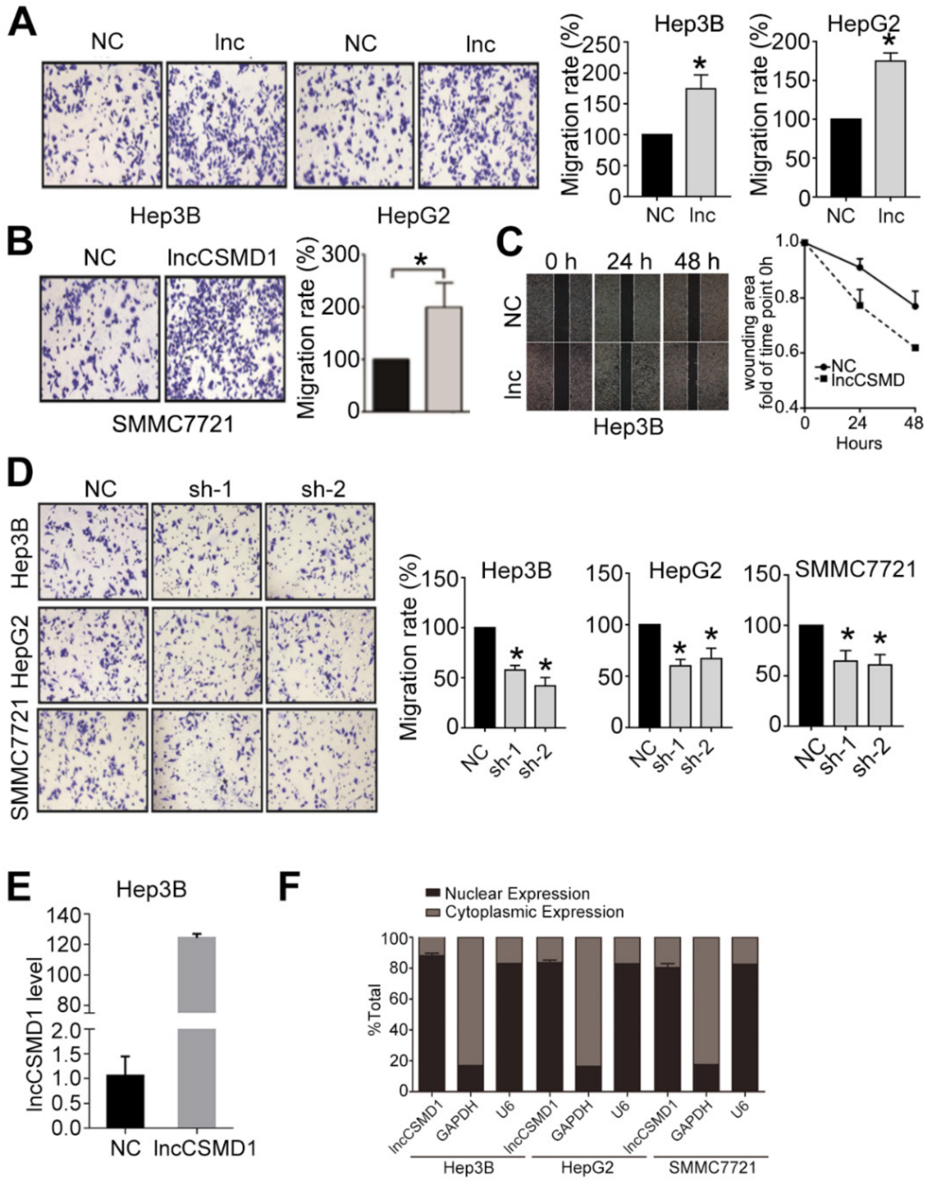

Figure S3. lncCSMD1 expression affects the migration of HCC cells. (**A, B**) Ectopic expression of lncCSMD1 facilitates the migration of Hep3B, HepG2 and SMMC7721 cells, as shown by the transwell assay. (**C**) Wound scratch assay indicates that lncCSMD1 expression can promote the migration of Hep3B cells. (**D**) Knockdown of lncCSMD1 inhibits the migration of Hep3B, HepG2 and SMMC7721 cells, as shown by the transwell assay. (**E**) The expression of lncCSMD1 was markedly elevated in xenograft tumor derived from Hep3B cells overexpressing lncCSMD1 compared with that in the tumor from the control cells, which was detected by RT-qPCR. (**F**) The vast majority of lncCSMD1 transcripts are located in the nucleus of HCC cells and only a few are in cytoplasm, in which qRT-PCR was

used to measure lncCSMD1 expression in the RNAs extracted from cytoplasm and nucleus, respectively. Data represent the mean ± SD of triplicate experiments and was analyzed by Student t test. *p < 0.05.

A

GENE SETS	SIZE	ES
HALLMARK_G2M_CHECKPOINT	154	0.66824
HALLMARK_E2F_TARGETS	157	0.61714
HALLMARK_MITOTIC_SPINDLE	145	0.59637
HALLMARK_MYC_TARGETS_V1	157	0.49055
HALLMARK_FATTY_ACID_METABOLISM	134	0.43156
HALLMARK_MTORC1_SIGNALING	160	0.39765
HALLMARK_ADIPOGENESIS	154	0.36161
HALLMARK_DNA_REPAIR	116	0.35941

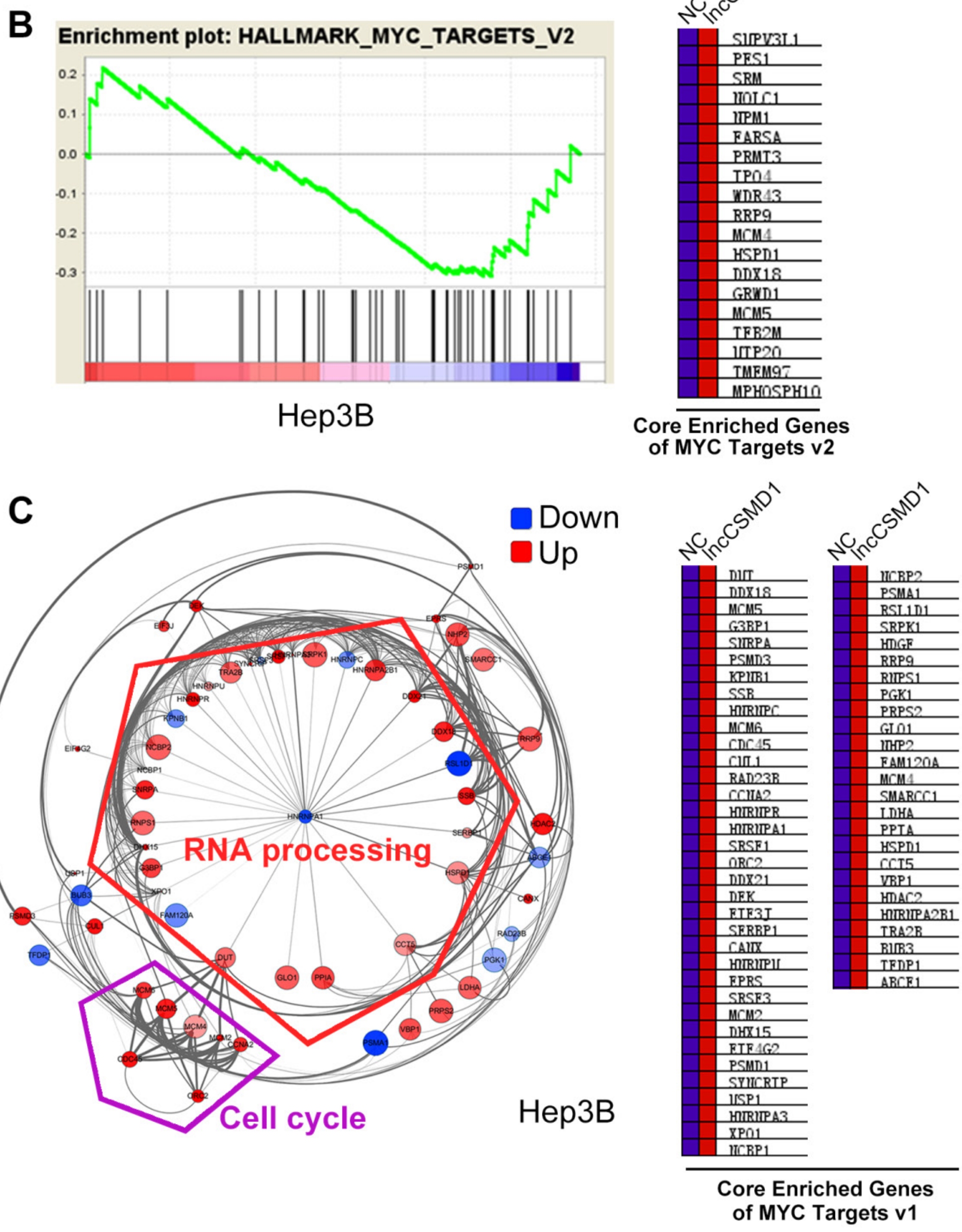

Figure S4. lncCSMD1 overexpression significantly enriched Myc target genes in HCC cells. (A) GSEA analysis revealed lncCSMD1 overexpression could markedly enriched several gene sets including Myc targets v1, which was involved in regulating proliferation of HCC cells; (**B**) Myc targets v2 were also enriched by lncCSMD1 overexpression. (**C**) The protein-protein interaction (PPI) network analysis shows the interaction of Myc target genes in set v1.

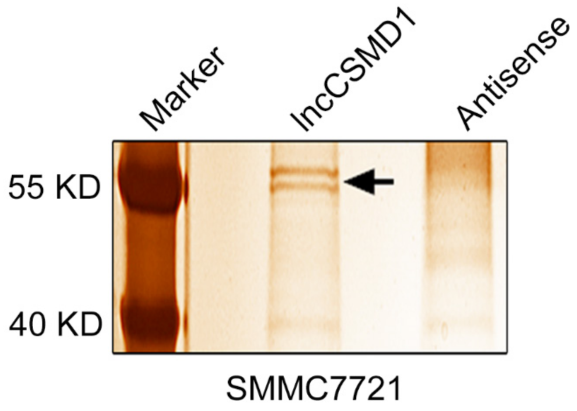

Figure S5. The specific band pulled down by lncCSMD1 in SMMC7721 cells. Silver staining of the precipitated complexes pulled down by lncCSMD1 sense and antisense probes on the PAGE gel, and a special bind (arrow) can be observed in the lane of sense probe.

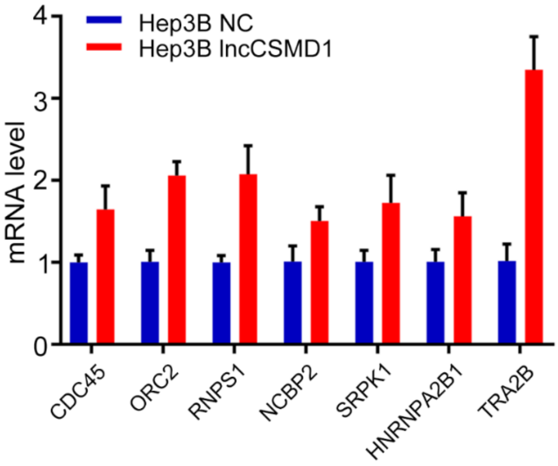

Figure S6. lncCSMD1 overexpression could activate the transcription of MYC target genes. qRT-PCR was used to examine the mRNA level of Myc target genes in Hep3B cells with lncCSMD1 overexpression or negative vector.

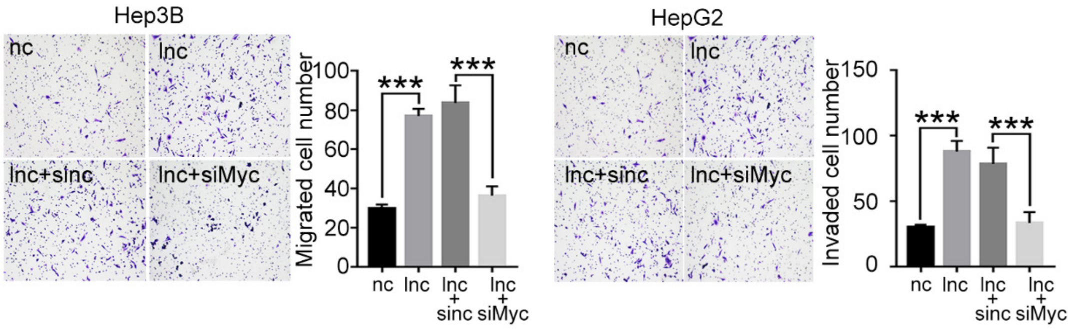

Figure S7. The knock-down of MYC could abolish the enhanced migration induced by lncCSMD1 overexpression. MYC was knocked down by siRNAs in Hep3B and HepG2 cells with overexpression of lncCSMD1, and then the migration ability was detected with transwell assay. The result shows that downregulated MYC completely abolishes the enhanced cell migration induced by lncCSMD1 overexpression compared with lncCSMD1 overexpression alone or plus siRNA control treatment in Hep3B and HepG2 cells.

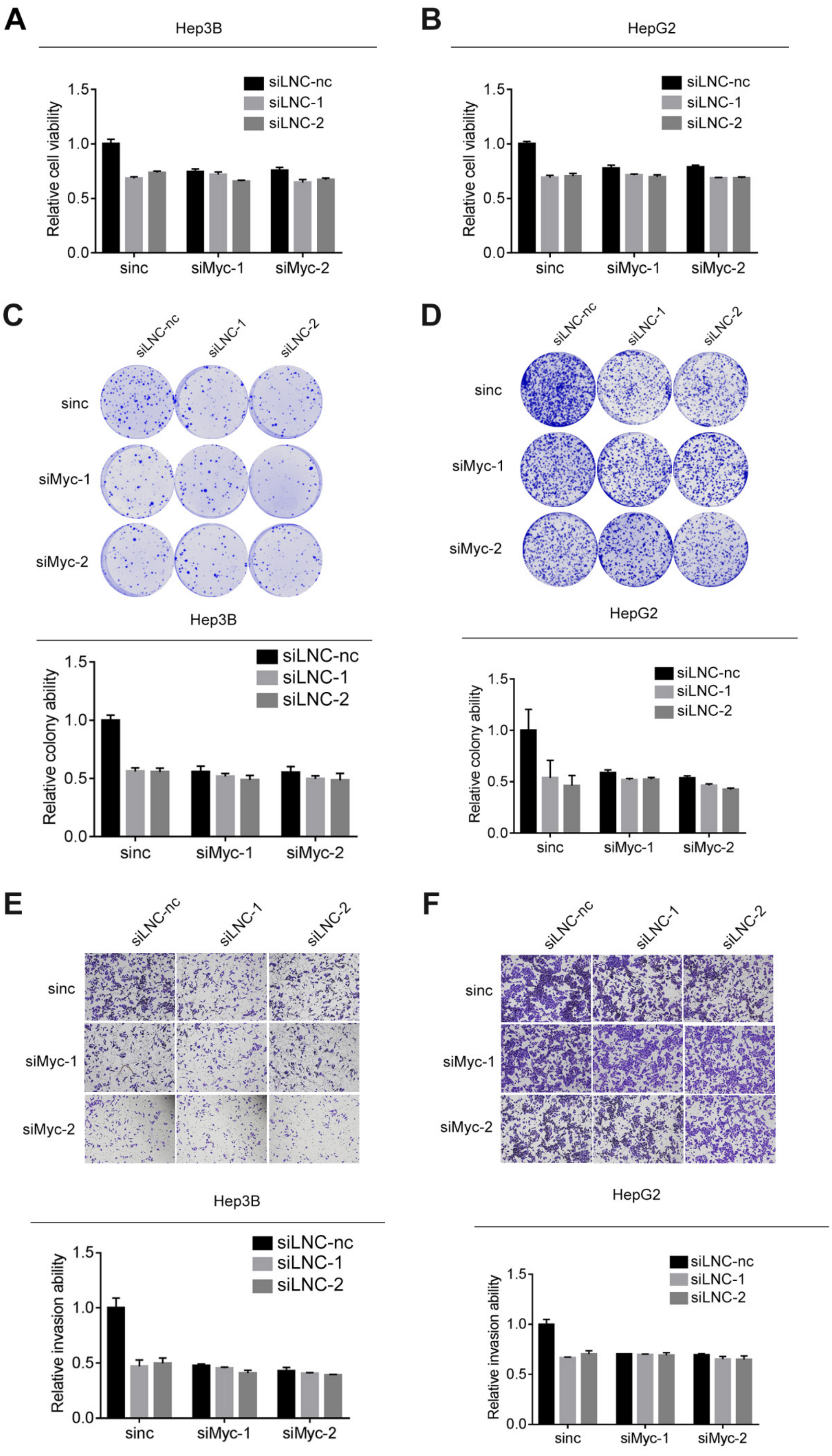

A
Hep3B
Relative cell viability
siLNC-nc
siLNC-1
siLNC-2
sinc
siMyc-1
siMyc-2
B
HepG2
Relative cell viability
C
siLNC-nc
siLNC-1
siLNC-2
sinc
siMyc-1
siMyc-2
Hep3B
Relative colony ability
D
HepG2
Relative colony ability
E
Hep3B
Relative invasion ability
F
HepG2
Relative invasion ability

Figure S8. The knock-down of LncCSMD1 failed to inhibit oncogenic phenotypes of HCC cells with downregulation of MYC. After lncCSMD1 was downregulated for 48 hours by siRNA in Hep3B and HepG2 cells with MYC knockdown by siRNA, CCK8 (A, B), colony formation (C, E) and invasion assays (D, F) were conducted to evaluate the effect of knockdown lncCSMD1 on oncogenic phenotypes of the HCC cells. The results demonstrate that lncCSMD1 knockdown cannot further affect the oncogenic phenotypes inhibited by MYC downregulation in Hep3B and HepG2 cells, indicating that lncCSMD1 promotes the oncogenic phenotypes via activating MYC signaling pathway.

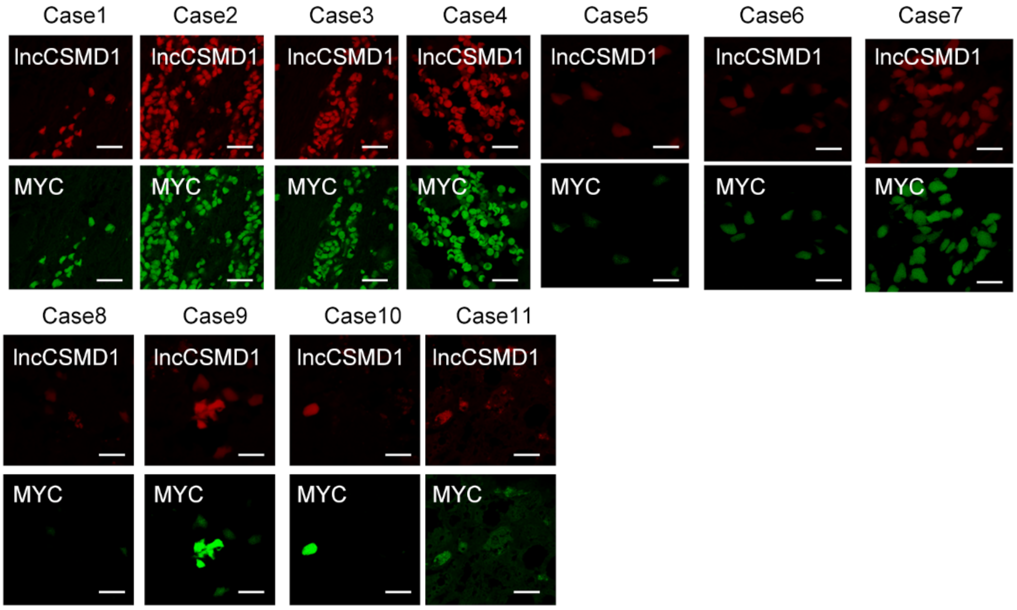

Figure S9. The correlation between lncCSMD1 and MYC protein level in 11 HCC tissue samples. LncCSMD1 RNA and Myc protein in 11 primary HCC tissue samples were simultaneously detected by RNA FISH (for lncCSMD1) and immunofluorescence, respectively, and a positive correlation between lncCSMD1 and MYC protein was observed.

2. SUPPLEMENTAL TABLES

Table S1. Relationship between lncCSMD1 level and the clinical characteristics of HCC patients in the discovery cohort (n=127).

Characteristics	lncCSMD1 intensity		
	Low intensity	high intensity	*P*-value
**Age(years)**			
≥50	38 (59.4%)	28 (44.4%)	0.110
<50	26 (40.6%)	35 (55.6%)	
**Gender**			
Female	8 (12.5%)	8 (12.7%)	1.000
Male	56 (87.5%)	55 (87.3%)	
**AFP (μg/L)**			
>400	42 (65.6%)	34 (54.0%)	0.210
≤400	22 (34.4%)	29 (46.0%)	
**HBsAg**			
Negative	11 (17.2%)	3 (4.8%)	**0.044**
Positive	53 (82.8%)	60 (95.2%)	
**HBV DNA**			
Negative	26 (40.6%)	15 (23.8%)	0.058
Positive	38 (59.4%)	48 (76.2%)	
**Pathology Grade**			
I	3 (4.7%)	0 (0.0%)	0.260
II	46 (71.9%)	45 (71.4%)	
III	15 (23.4%)	18 (28.6%)	
**Cirrhosis**			
NO	33 (51.6%)	20 (31.7%)	**0.031**
Yes	31 (48.4%)	43 (68.3%)	
**Main Size**			
<5cm	32 (50.0%)	24 (38.1%)	0.210
≥5cm	32 (50.0%)	39 (61.9%)	
**Tumor Number**			
1	54 (84.4%)	33 (52.4%)	**< 0.001**
≥2	10 (15.6%)	30 (47.6%)	
**Tumor capsule**			
No	18 (28.1%)	25 (39.7%)	0.190
Yes	46 (71.9%)	38 (60.3%)	
**Cancer Embolus**			
No	54 (84.4%)	48 (76.2%)	0.270
Yes	10 (15.6%)	15 (23.8%)	
**TNM stage**			
I	44 (68.8%)	24 (38.1%)	**< 0.001**
II	13 (20.3%)	15 (23.8%)	
III	7 (10.9%)	24 (38.1%)	
**Metastasis**			
No	59 (92.2%)	45 (71.4%)	**0.003**
Yes	5 (7.8%)	18 (28.6%)	
**Relapse**			
No	45 (70.3%)	37 (58.7%)	0.200
Yes	19 (29.7%)	26 (41.3%)	

Table S2. Relationship between lncCSMD1 level and the clinical characteristics of HCC patients in the validation cohort (n=260).

Characteristics	lncCSMD1 level		
	low expression	high expression	*P*-value
**Age(years)**			
≥50	66 (50.8%)	68 (52.3%)	0.900
<50	64 (49.2%)	62 (47.7%)	
**Gender**			
Female	18 (13.8%)	17 (13.1%)	1.000
Male	112 (86.2%)	113 (86.9%)	
**AFP (μg/L)**			
>400	88 (67.7%)	83 (63.8%)	0.600
≤400	42 (32.3%)	47 (36.2%)	
**HBsAg**			
Negative	20 (15.4%)	7 (5.4%)	**0.013**
Positive	110 (84.6%)	123 (94.6%)	
**HBV DNA**			
Negative	40 (30.8%)	33 (25.4%)	0.410
Positive	90 (69.2%)	97 (74.6%)	
**Pathology Grade**			
I	8 (6.2%)	9 (6.9%)	0.880
II	84 (64.6%)	80 (61.5%)	
III	38 (29.2%)	41 (31.5%)	
**Cirrhosis**			
NO	55 (42.3%)	53 (40.8%)	0.900
Yes	75 (57.7%)	77 (59.2%)	
**Main Size**			
<5cm	50 (38.5%)	53 (40.8%)	0.800
≥5cm	80 (61.5%)	77 (59.2%)	
**Tumor Number**			
1	91 (70.0%)	81 (62.3%)	0.24
≥2	39 (30.0%)	49 (37.7%)	
**Tumor capsule**			
No	43 (33.1%)	48 (36.9%)	0.600
Yes	87 (66.9%)	82 (63.1%)	
**Cancer Embolus**			
No	95 (73.1%)	100 (76.9%)	0.570
Yes	35 (26.9%)	30 (23.1%)	
**TNM stage**			
I	58 (44.6%)	53 (40.8%)	0.640
II	35 (26.9%)	42 (32.3%)	
III	37 (28.5%)	35 (26.9%)	
**Metastasis**			
No	116 (89.2%)	110 (84.6%)	0.360
Yes	14 (10.8%)	20 (15.4%)	
**Relapse**			
No	86 (66.2%)	74 (56.9%)	0.160
Yes	44 (33.8%)	56 (43.1%)	

Table S3. Relationship between lncCSMD1 expression and the clinical characteristics of HCC patients in external validation cohort 1 (n=92).

Characteristics	lncCSMD1 level		
	Low expression	high expression	*P*-value
**Age(years)**			
≥50	10 (21.7%)	5 (10.9%)	0.260
<50	36 (78.3%)	41 (89.1%)	
**Gender**			
Female	33 (71.7%)	33 (71.7%)	1.000
Male	13 (28.3%)	13 (28.3%)	
**AFP (μg/L)**			
>400	28 (60.9%)	29 (63.0%)	1.000
≤400	18 (39.1%)	17 (37.0%)	
**HBsAg**			
Negative	9 (26.5%)	14 (32.6%)	0.620
Positive	25 (73.5%)	29 (67.4%)	
**HBV DNA**			
Negative	20 (50.0%)	9 (21.4%)	**0.011**
Positive	20 (50.0%)	33 (78.6%)	
**Pathology Grade**			
I	14 (30.4%)	4 (9.8%)	**< 0.001**
II	27 (58.7%)	19 (46.3%)	
III	5 (10.9%)	18 (43.9%)	
**Cirrhosis**			
NO	8 (17.4%)	6 (13.0%)	0.770
Yes	38 (82.6%)	40 (87.0%)	
**Main Size**			
<5cm	31 (67.4%)	17 (37.0%)	**0.006**
≥5cm	15 (32.6%)	29 (63.0%)	
**Tumor Number**			
1	45 (97.8%)	25 (54.3%)	**< 0.001**
≥2	1 (2.2%)	21 (45.7%)	
**Tumor capsule**			
No	40 (87.0%)	30 (65.2%)	**0.026**
Yes	6 (13.0%)	16 (34.8%)	
**Cancer Embolus**			
No	5 (10.9%)	18 (39.1%)	**0.003**
Yes	41 (89.1%)	28 (60.9%)	
**TNM stage**			
I	33 (71.7%)	10 (21.7%)	**< 0.001**
II	0 (0.0%)	10 (21.7%)	
III	13 (28.3%)	26 (56.5%)	

Table S4. Relationship between lncCSMD1 expression and the clinical characteristics of HCC patients of external cohort 2 (n=124).

Characteristics	lncCSMD1 level		
	Low expression	high expression	*P*-value
**Age(years)**			
≥50	4 (6.5%)	5 (8.1%)	1.000
<50	58 (93.5%)	57 (91.9%)	
**Gender**			
Female	40 (64.5%)	36 (58.1%)	0.580
Male	22 (35.5%)	26 (41.9%)	
**AFP (μg/L)**			
>400	40 (64.5%)	35 (56.5%)	0.460
≤400	22 (35.5%)	27 (43.5%)	
**HBsAg**			
Negative	23 (37.1%)	24 (38.7%)	1.000
Positive	39 (62.9%)	38 (61.3%)	
**HBV DNA**			
Negative	27 (45.8%)	9 (16.4%)	**0.001**
Positive	32 (54.2%)	46 (83.6%)	
**Pathology Grade**			
I	2 (4.1%)	1 (7.1%)	0.350
II	12 (24.5%)	1 (7.1%)	
III	35 (71.4%)	12 (85.7%)	
**Cirrhosis**			
NO	8 (12.9%)	6 (9.7%)	0.780
Yes	54 (87.1%)	56 (90.3%)	
**Main Size**			
<5cm	35 (56.5%)	20 (32.3%)	**0.011**
≥5cm	27 (43.5%)	42 (67.7%)	
**Tumor Number**			
1	37 (59.7%)	4 (6.5%)	**< 0.001**
≥2	25 (40.3%)	58 (93.5%)	
**Tumor capsule**			
No	16 (25.8%)	40 (64.5%)	**< 0.001**
Yes	46 (74.2%)	22 (35.5%)	
**Cancer Embolus**			
No	14 (22.6%)	50 (80.6%)	**< 0.001**
Yes	48 (77.4%)	12 (19.4%)	
**TNM stage**			
I	11 (17.7%)	2 (3.2%)	**0.006**
II	24 (38.7%)	18 (29.0%)	
III	27 (43.5%)	42 (67.7%)	

Table S5. Multivariate Cox analysis of lncCSMD1 expression and clinical characteristics associated with survival in the Discovery and Validation cohorts.

Characteristics	Discovery cohort N=127		Validation cohort N=260	
	HR (95% CI)	*P* value	HR (95% CI)	*P* value
**Overall survival**				
lncCSMD1	1.51 (0.81-2.81)	0.180	1.67 (1.12-2.51)	**0.013**
AFP	1.22 (0.67-2.22)	0.500		
Pathology Grade			1.18 (0.80-1.74)	0.402
Cirrhosis	1.53 (0.85-2.74)	0.140	1.12 (0.76-1.67)	0.560
Main.Size	1.50 (0.83-2.71)	0.170		
Tumor Number	1.07 (0.45-2.57)	0.860	1.58 (1.03-2.43)	**0.038**
Tumor Capsule	0.73 (0.42-1.27)	0.270	0.70 (0.46-1.08)	0.110
Cancer Embolus	1.47 (0.74-2.89)	0.261	1.29 (0.69-2.41)	0.427
TNM Stage	2.13 (1.23-3.69)	**0.006**	1.31 (0.91-1.88)	0.145
Metastasis	0.94 (0.51-1.75)	0.867	3.11 (1.97-4.92)	**<0.001**
Relapse	1.79 (1.06-3.01)	**0.028**	1.92 (1.31-2.83)	**<0.001**
**Disease free survival**				
lncCSMD1	1.17 (0.68-1.99)	0.568	1.68 (1.12-2.52)	**0.013**
AFP	1.60 (0.93-2.75)	0.090	1.10 (0.74-1.63)	0.650
Pathology Grade			1.13 (0.77-1.65)	0.543
Cirrhosis	1.65 (0.95-2.86)	0.074		
Main Size	1.47 (0.73-2.95)	0.282	1.47 (0.84-2.57)	0.179
Tumor Number	1.19 (0.51-2.77)	0.686	1.88 (1.21-2.93)	**0.005**
Tumor Capsule	0.64 (0.38-1.08)	0.093		
Cancer Embolus	1.41 (0.52-3.81)	0.497	1.45 (0.77-2.72)	0.247
TNM Stage	1.87 (1.11-3.16)	**0.019**	1.01 (0.66-1.56)	0.953
Metastasis	1.54 (0.83-2.87)	0.173	2.74 (1.73-4.34)	**<0.001**
Relapse	5.32 (3.19-8.87)	**<0.001**	1.86 (1.26-2.75)	**0.0017**

Table S6. Multivariate Cox analysis of lncCSMD1 expression and clinical characteristics associated with survival in the two External validation cohorts.

Characteristics	Ext Valid Cohort 1 N=92		Ext Valid Cohort 2 N=124	
	HR (95%CI)	*P* value	HR (95% CI)	*P* value
**Overall survival**				
lncCSMD1	4.85 (1.37-17.11)	**0.014**	9.05 (1.85-44)	**0.007**
AFP				
Pathology Grade	2.43 (1.29-4.58)	**0.006**	2.78 (0.38-20)	0.317
Cirrhosis				
Main Size	11.0 (1.56-77.9)	**0.016**	0.003 (0.0-Inf)	0.998
Tumor Number	1.92 (0.56-6.63)	0.300	2.73 (0.22-33.9)	0.435
Tumor Capsule	0.50 (0.20-1.26)	0.140		
Cancer Embolus	2.50 (0.77-8.10)	0.126	1.04 (0.1-11.4)	0.973
TNM stage	0.21 (0.06-0.67)	**0.009**	454 (0.00-Inf)	0.998
**Disease free survival**				
lncCSMD1	1.57 (0.64-3.84)	0.326	9.06 (1.84-44.5)	**0.007**
Pathology Grade	1.75 (1.06-2.91)	**0.030**	2.88 (0.39-21.2)	0.298
Tumor Number	0.65 (0.27-1.52)	0.319	2.83 (0.23-34.8)	0.415
Tumor Capsule	0.87 (0.40-1.89)	0.731		
Cancer Embolus	0.66 (0.29-1.50)	0.319	1.09 (0.10-11.6)	0.946
TNM stage			1.61 (0.73-3.56)	0.242

Table S7. Univariate Cox analysis of lncCSMD1 expression and clinical characteristics associated with survival in all patients of the four cohorts.

Characteristics	All patients N=603	
	**HR (95%CI)**	**P value**
**Overall survival**		
lncCSMD1	3.80 (2.80-5.00)	**<0.001**
Age	0.75 (0.58-0.96)	**0.025**
Gender	1.30 (0.99-1.70)	0.055
AFP	1.60 (1.30-2.10)	**<0.001**
Pathology Grade	1.50 (1.20-1.70)	**<0.001**
Cirrhosis	1.20 (0.89-1.50)	0.260
Main Size	2.30 (1.70-3.00)	**<0.001**
Tumor Number	2.80 (2.20-3.60)	**<0.001**
Tumor capsule	1.00 (0.80-1.30)	0.800
Cancer Embolus	0.84 (0.64-1.10)	0.190
TNM stage	1.70 (1.50-2.00)	**<0.001**
**Disease free survival**		
lncCSMD1	2.70 (2.10-3.40)	**<0.001**
Age	0.65 (0.52-0.81)	**<0.001**
Gender	1.50 (1.20-1.90)	**0.001**
AFP	1.60 (1.30-2.00)	**<0.001**
Pathology Grade	1.20 (1.00-1.40)	**0.009**
Cirrhosis	0.98 (0.78-1.20)	0.900
Main Size	1.80 (1.50-2.30)	**<0.001**
Tumor Number	2.10 (1.70-2.60)	**<0.001**
Tumor capsule	1.10 (0.85-1.30)	0.590
Cancer Embolus	0.76 (0.60-0.96)	**0.021**
TNM stage	1.40 (1.20-1.60)	**<0.001**

Table S8. Multivariate Cox analysis of lncCSMD1 expression and clinical characteristics associated with survival in all patients of the four cohorts.

Characteristics	All patients N=603	
	HR (95%CI)	P value
**Overall survival**		
lncCSMD1	2.76 (2.03-3.74)	**<0.001**
Age	0.67 (0.52-0.88)	**0.004**
AFP	1.21 (0.94-1.57)	0.132
Pathology Grade	1.04 (0.88-1.24)	0.607
Main Size	1.86 (1.31-2.64)	**0.001**
Tumor Number	2.01 (1.45-2.78)	**<0.001**
TNM stage	1.06 (0.85-1.32)	0.578
**Disease free survival**		
lncCSMD1	2.21 (1.72-2.84)	**<0.001**
Age	0.63 (0.50-0.79)	**<0.001**
Gender	1.44 (1.11-1.88)	**0.006**
AFP	1.32 (1.05-1.66)	**0.016**
Pathology Grade	0.96 (0.82-1.13)	0.673
Main Size	1.68 (1.22-2.32)	**0.001**
Tumor Number	1.83 (1.36-2.46)	**<0.001**
Cancer Embolus	1.06 (0.78-1.43)	0.684
TNM stage	0.99 (0.79-1.24)	0.957

Table S9. Primers used in this study.

**Primers for q-PCR**	Sequences
GAPDH Forward	CTCCTCCTGTTCGACAGTCAGC
GAPDH Reverse	CCCAATACGACCAAATCCGTT
lncCSMD1 Forward	CAACCCACGCCCATGAATCT
lncCSMD1 Reverse	AGTGGCAGGGACGACAATG
Myc Forward	TCCACCTCCAGCTTGTACCT
Myc Reverse	GCTGTCGTTGAGAGGGTAGG
E2F Forward	CATCCCAGGAGGTCACTTCTG
E2F Reverse	GACAACAGCGGTTCTTGCTC
UTP20 Forward	GCAGATGGATTTCTACCCACAC
UTP20 Reverse	GAGGTGAAAGCCCATTCTAACAA
RRP9 Forward	GGACTGTACGTGTGTGGAAGA
RRP9 Reverse	CTCCTCATTGATTAGGTGGATGC
DHX15 Forward	GGGGACCGATGGGAAGGAT
DHX15 Reverse	TAGCATTTGTTGAAGCTCGCA
MCM5 Forward	AGCATTCGTAGCCTGAAGTCG
MCM5 Reverse	CGGCACTGGATAGAGATGCG
DDX21 Forward	CTTTGCCATCCCTTTGATTGAGA
DDX21 Reverse	GTAGGTGCAAGAACCAGTACC
CDC45 Forward	CTTGAAGTTCCCGCCTATGAAG
CDC45 Reverse	GCATGGTTTGCTCCACTATCTC
ORC2 Forward	AGAGCTAAACTGGATCAGCAAAC
ORC2 Reverse	CACAATGTTGAACCCAAGGTGTA
RNPS1 Forward	AGAGCTTGCTAGGAGTCAAAGA
RNPS1 Reverse	TTGAGCGATCCTTGGACTTCT
NCBP2 Forward	AAAACGCCATGCGGTACATAA
NCBP2 Reverse	GCCTGCCCTCCTTAAAGCC
SRPK1 Forward	ATGGAGCGGAAAGTGCTTG
SRPK1 Reverse	GAGCCTCGGTGCTGAGTTT
HNRNPA2B1 Forward	ATTGATGGGAGAGTAGTTGAGCC
HNRNPA2B1 Reverse	AATTCCGCCAACAAACAGCTT
TRA2B Forward	GCAGTCTAGGCGTTCAAGAGG
TRA2B Reverse	CATTGGCACGTTCTTTAGCTTC
U6 Forward	CTCGCTTCGGCAGCACA
U6 Reverse	AACGCTTCACGAATTTGCGT
**LncCSMD1 siRNA:**	
lncCSMD1 - si#1 target sequence	CCTCCTGATCTGCAGCATT
lncCSMD1 - si#2 target sequence	GCGGATGAAGACATTGATG
**Myc siRNA:**	
Myc- si#1 target sequence	CCTGAGACAGATCAGCAACAA
**Control siRNA:**	
Myc - si#NC Forward	GAGGCGAAGGATGACAAAGGGA
Myc - si#NC Reverse	GACAGATGTCCAGCCACAATTCT
